# Nanoparticulate and Hydrogel Vehicles for Stimuli-Responsive and Sustained Controlled Release of Active Pharmaceutical Ingredients

**DOI:** 10.3390/ph19081324

**Published:** 2026-08-21

**Authors:** Simona Ardelean, Ioana Ciopănoiu, Ioana Cuc-Hepcal, Anda O. J. Samoila, Corina Morodan, Mihaela Borlea, Silviu L. Constantinescu, Oana Koppandi, Sorina Ciurlea, Carmen Tomoroga, Adriana Ledeți, Livia C. Borcan, George A. Drăghici, Paul Albu, Cristina A. Dehelean

**Affiliations:** 1 Faculty of Pharmacy, Vasile Goldiș Western University of Arad, 86 L. Rebreanu St., 310414 Arad, Romania; ardelean.simona@uvvg.ro (S.A.); ciopanoiu.ioana@uvvg.ro (I.C.); hepcal.ioana@uvvg.ro (I.C.-H.); samoila.anda@uvvg.ro (A.O.J.S.); morodan.corina@uvvg.ro (C.M.); borlea.mihaela@uvvg.ro (M.B.); ciurlea.sorina@uvvg.ro (S.C.); 2 Multidisciplinary Doctoral School, Vasile Goldiș Western University of Arad, 86 L. Rebreanu St., 310414 Arad, Romania; constantinescu.silviu-laurentiu@student.uvvg.ro (S.L.C.); koppandi.oana@uvvg.ro (O.K.); 3 Department of Analytical Chemistry, Faculty of Pharmacy, “Victor Babeș” University of Medicine and Pharmacy Timișoara, Eftimie Murgu Square No. 2, 300041 Timișoara, Romania; axente.carmen@umft.ro (C.T.); afulias@umft.ro (A.L.); 4 Advanced Instrumental Screening Center, Faculty of Pharmacy, “Victor Babeş” University of Medicine and Pharmacy Timișoara, Eftimie Murgu Square No. 2, 300041 Timișoara, Romania; 5 Department V (Internal Medicine I), Faculty of Medicine, “Victor Babeș” University of Medicine and Pharmacy Timișoara, 12 Revoluției din 1989 Blvd., 300041 Timișoara, Romania; borcan.cristina@umft.ro; 6 Faculty of Pharmacy, “Victor Babes” University of Medicine and Pharmacy Timisoara, Eftimie Murgu Square, No. 2, 300041 Timisoara, Romania; draghici.george-andrei@umft.ro (G.A.D.); cadehelean@umft.ro (C.A.D.); 7 Research Center for Pharmaco-Toxicological Evaluations, Faculty of Pharmacy, “Victor Babes” University of Medicine and Pharmacy Timisoara, Eftimie Murgu Square, No. 2, 300041 Timisoara, Romania

**Keywords:** nanoparticles, hydrogels, stimuli-responsive drug delivery, sustained release, controlled release, tumor microenvironment, injectable depots, machine learning, clinical translation

## Abstract

Most active pharmaceutical ingredients (APIs) reach their target by passive systemic distribution, so the dose required for efficacy at the lesion is set by what healthy tissue can tolerate; conventional dosage forms consequently produce pharmacokinetic profiles that oscillate between toxic peaks and sub-therapeutic troughs. Nanoparticulate carriers (liposomes, lipid nanoparticles, polymeric and inorganic systems, and biomimetic carriers) and hydrogels (natural, synthetic, supramolecular, and microgel-assembled) have emerged as the dominant strategies to address this, increasingly combined as hybrid nanoparticle–hydrogel constructs in which the gel provides locoregional retention and the nanoparticles provide cargo protection, intracellular delivery and stimuli responsiveness. Stimuli-responsive chemistries (pH, redox, enzyme, ROS, hypoxia, temperature, light, magnetic, ultrasound, glucose, and multi-stimuli logic) translate the molecular signatures of a disease into spatiotemporally controlled cargo release. This narrative review consolidates the state of the art (prioritizing 2022–2026) and departs from the conventional carrier-type survey in one respect: the literature is read along an explicit chain—disease cue, sensing chemistry, carrier architecture, release mechanism and kinetics, administration route, and clinical readiness—which exposes a variable that classification by carrier type conceals. Across all three material classes, what governs release behavior is not primarily the carrier chemistry but the identity of the released species (dissolved drug, drug from an embedded nanoparticle, an intact nanoparticle, and a matrix fragment) and the transport step that limits it. This is why power-law exponent analysis developed for dissolved drug fits particulate release poorly, why statistical goodness-of-fit cannot by itself establish a release mechanism, and why carrier class predicts clinical readiness less well than administration route and regulatory product type. Translational hurdles—CMC complexity, regulatory fragmentation, anti-PEG immunogenicity, and the structural mismatch between preclinical promise and clinical efficacy—are critically appraised in light of previously reported <1% delivery efficiency analysis. This review identifies converging strategies that could move stimuli-responsive controlled release from an aspirational outcome to a routine clinical reality.

## 1. Introduction

Despite more than a century of pharmaceutical innovation, the majority of clinically administered active pharmaceutical ingredients (APIs) still rely on dosage forms whose pharmacokinetic behavior is governed by passive, systemic distribution. Repeated oral or parenteral dosing inevitably produces oscillations between supra-therapeutic peaks—which expose healthy tissues to toxic concentrations—and sub-therapeutic troughs, during which efficacy is lost and, in the case of antimicrobials, resistance can be selected for [1,2]. These limitations are particularly acute for drugs with narrow therapeutic indices, poor aqueous solubility, short biological half-lives, or restricted access to anatomically privileged sites, such as the central nervous system, the posterior segment of the eye and avascular tumor cores [3,4]. The conceptual response to these problems has been progressive: from sustained release formulations that prolong drug exposure, to controlled release systems that aim to maintain therapeutic concentrations within a defined window, to the contemporary paradigm of stimuli-responsive or “smart” delivery, in which the carrier itself senses a pathological cue and discharges its cargo on demand [3,5,6].

Two material platforms have come to dominate this evolution. Nanoparticulate vehicles—liposomes, lipid nanoparticles (LNPs), polymeric nanoparticles, dendrimers, micelles, mesoporous silica, iron oxide and other inorganic carriers—exploit nanoscale dimensions to extend circulation, leverage passive (enhanced permeability and retention (EPR)) or active targeting, and protect labile cargoes from premature degradation [4,7,8]. Their clinical track record now includes more than a dozen approved liposomal and lipid-based products, and the rapid global deployment of mRNA–LNP vaccines has demonstrated that nanocarriers can be manufactured, regulated and distributed at the industrial scale [7,9]. Yet, despite these successes, a recent systematic review of nanocarrier studies between 2016 and April 2025 confirmed that translational efficiency remains modest: the majority of nanomedicines still fail at the bench-to-bedside interface, with rapid mononuclear phagocyte clearance, low tumor accumulation in patients, batch-to-batch variability and chemistry, manufacturing and controls (CMC) complexity repeatedly identified as the dominant obstacles [9,10].

Hydrogels—three-dimensional, hydrophilic, polymeric networks capable of imbibing large amounts of biological fluid while preserving structural integrity—provide a complementary solution. Their high biocompatibility, soft-tissue-like mechanics, extracellular matrix mimicry and ease of local administration via injection, implantation or topical application make them ideal depots for prolonged, locoregional release [11,12,13]. The transition from “dumb” to “smart” hydrogels, capable of altering swelling, mesh size, hydrophilicity or chain conformation in response to physical or biochemical cues, has accelerated dramatically over the past five years, with thousands of peer-reviewed studies published annually [12,14]. Nevertheless, bulk hydrogels alone struggle to encapsulate hydrophobic APIs, often suffer from initial burst release, and are limited in their ability to deliver finely tuned, multi-phasic kinetics [11,15].

These complementary strengths and weaknesses have driven a strong recent convergence: the embedding of nanoparticles within hydrogel matrices to produce nanocomposite or nanoparticle–hydrogel hybrid systems [11,15,16,17]. In such constructs, the hydrogel acts as a retentive, biocompatible reservoir that suppresses burst release and enables local administration, while the nanoparticles serve as compartments that solubilize hydrophobic drugs, protect biologics, fine-tune release kinetics, confer stimuli-responsiveness and provide targeting capability after eventual release from the matrix [11,15,18]. Recent comprehensive reviews have highlighted that such hybrids can simultaneously achieve sustained release of weeks to months, programmable multi-drug release sequences, and on-demand triggering by external or pathological cues [11,15,17].

The diversity of stimuli now exploited reflects both the progress of materials chemistry and a deeper understanding of disease microenvironments. Endogenous triggers—acidic pH in tumors, endo-lysosomes and infected tissues; elevated glutathione, reactive oxygen species and overexpressed enzymes (matrix metalloproteinases, hyaluronidases, and esterases); hypoxia; and abnormal glucose levels—are routinely used to switch on cargo release inside diseased cells or compartments [5,6,19,20,21]. Exogenous triggers—temperature, near-infrared (NIR) light, alternating magnetic fields, focused ultrasound and electric fields—provide spatiotemporal control by the operator, enabling drug release to be time-locked to imaging or treatment sessions and to be combined with hyperthermia, photodynamic, photothermal or sonodynamic therapy [6,22,23,24]. Multi-stimuli systems, which respond to two or more cues, and the subset of them that are genuinely AND-gated, which require the coincidence of both, are emerging as a strategy to further reduce off-target activation and improve specificity. The two terms are not synonymous; the distinction is developed in Section 6.9 [25,26].

Mechanistically, the design of these vehicles increasingly relies on quantitative description and prediction of release. Classical kinetic models—zero-order, first-order, Higuchi’s, Hixson–Crowell, Korsmeyer–Peppas, Peppas–Sahlin, and Weibull—remain indispensable for interpreting in vitro data and identifying whether release is governed by diffusion, polymer relaxation, swelling, surface erosion or bulk degradation [27,28,29]. More recently, machine learning- and data-driven approaches have begun to be layered on top of these physical models, allowing high-dimensional formulation spaces (polymer composition, crosslinking density, NP loading, and geometry) to be screened in silico and release profiles to be optimized prospectively for specific therapeutic windows [12,30].

Therapeutic applications now span the full clinical spectrum. In oncology, tumor-microenvironment-responsive nanocarriers and injectable depots are entering clinical evaluation for chemo–immunotherapy combinations, with TME pH, GSH, ROS and enzyme cues used to localize cytotoxic and immunomodulatory payloads [4,5,8,18]. In endocrinology, glucose-responsive insulin delivery—using glucose oxidase, phenylboronic acids or concanavalin A motifs in hydrogels, microgels and nanoparticles—is approaching the long-sought goal of a closed-loop, “artificial pancreas” pharmaceutical [21,31,32]. In regenerative medicine, infection and wound care, NP–hydrogel composites combining antimicrobial, pro-angiogenic and pro-resolving cues are being deployed as multifunctional dressings and injectable scaffolds [11,15]. In ocular delivery, polymer–nanoparticle (PNP) injectable hydrogels form slowly eroding intravitreal depots that keep a small-molecule glaucoma drug measurable in the vitreous for eight weeks after a single administration in rabbits [33].

Against this rich background, the present narrative review consolidates the state of the art on nanoparticulate and hydrogel vehicles for stimuli-responsive and sustained controlled release of APIs, with three deliberate emphases beyond a conventional materials survey: (i) the physico-chemical mechanisms underlying release and stimuli-responsiveness, including their mathematical description; (ii) the therapeutic applications in which these systems are most advanced, particularly oncology, diabetes, regenerative medicine and locoregional delivery; and (iii) the emerging frontiers of artificial intelligence-guided formulations, multi-stimuli logic, and biomimetic and theranostic platforms. The literature was selected by structured searches in PubMed, Web of Science, Scopus, the *Journal of Controlled Release*, *Advanced Drug Delivery Reviews*, *Nature Reviews Drug Discovery*, *Advanced Materials*, and the *ACS Nano and Biomaterials* archives, with priority given to studies published between 2022 and 2026; older works are cited only where historically necessary or where they remain the canonical reference for a specific concept.

**Figure 1:**
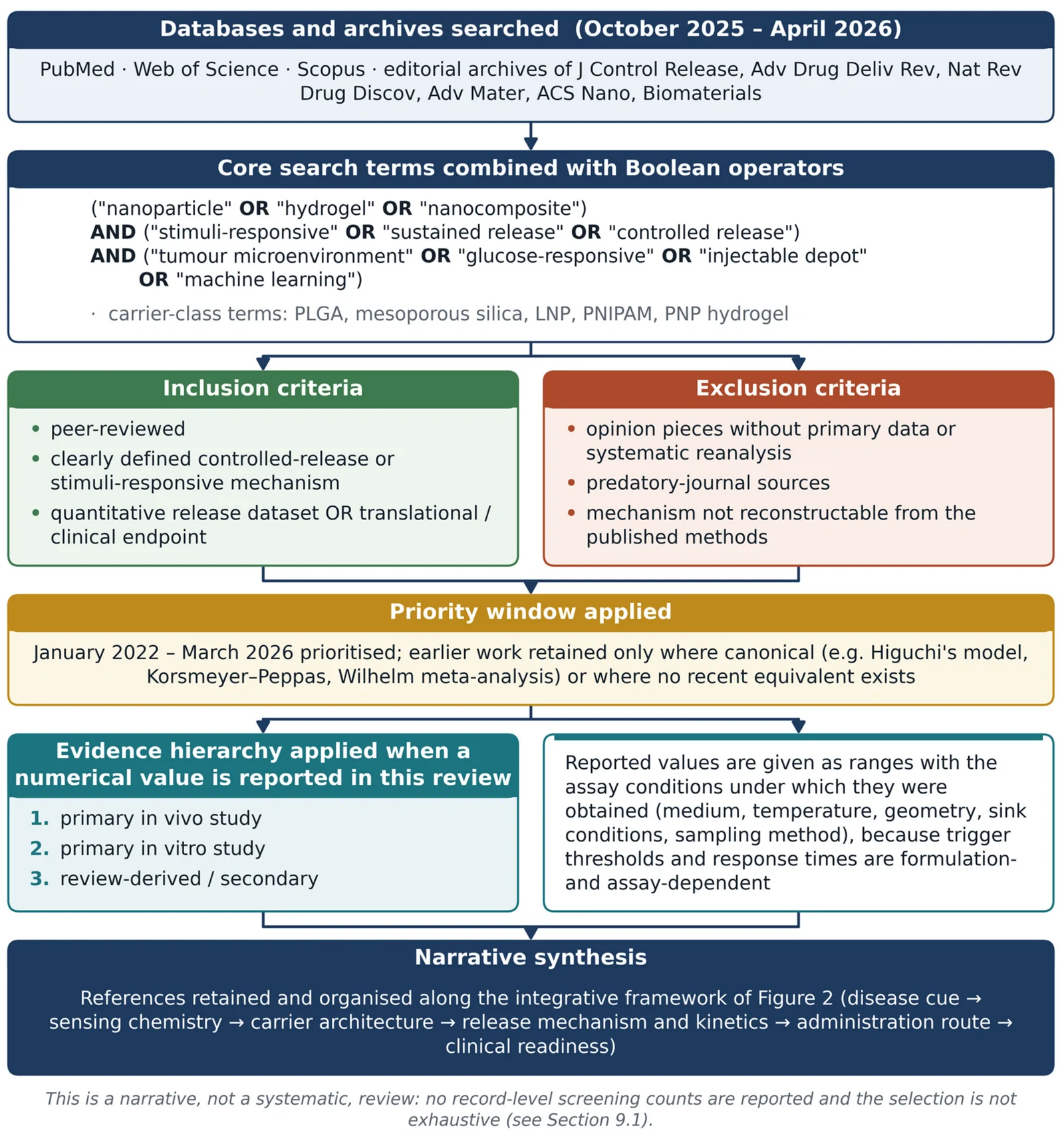
Literature search strategy, inclusion and exclusion criteria, and the evidence hierarchy applied when a numerical value is reported. The procedure is structured but not systematic: no record-level screening counts are reported (Section “Limitations of This Review”). Preprints and other non-peer-reviewed research reports were excluded; the small number of regulatory, manufacturer and trial registry documents cited are identified as such at the point of use (Section 2.1). Original figure created by the authors using Matplotlib 3.10.8 (Python 3.12.3).

The literature underpinning this review was assembled by structured searching rather than by systematic screening, and the procedure is set out in full in Figure 1 so that it can be reproduced. PubMed, Web of Science, Scopus and the editorial archives of the *Journal of Controlled Release*, *Advanced Drug Delivery Reviews*, *Nature Reviews Drug Discovery*, *Advanced Materials*, and *ACS Nano* and *Biomaterials* were searched between October 2025 and April 2026. Core terms were combined by Boolean operators, as shown in Figure 1, and supplemented with carrier-class terms (PLGA, mesoporous silica, LNP, PNIPAM, or PNP hydrogel). Priority was given to work published between January 2022 and March 2026; earlier work was retained only where it remained canonical or where no recent equivalent existed. Inclusion required peer review, a clearly defined controlled release or stimuli-responsive mechanism, and either a quantitative release dataset or a translational or clinical endpoint; preprints and other non-peer-reviewed research reports were not cited. Regulatory and manufacturer documents are cited in the small number of places where a regulatory or marketing status fact is itself the point at issue, and no peer-reviewed source records it, and are identified as such at the point of use. Studies were excluded if they were opinion pieces without primary data or systematic reanalysis, if they appeared in predatory outlets, or if the mechanism could not be reconstructed from the published methods.

Two reporting conventions follow from this procedure and are applied throughout. First, wherever a numerical value is quoted, preference is given to primary studies over secondary sources, and the evidence level is made explicit. Second, trigger thresholds and response times are reported as ranges together with the assay conditions under which they were obtained, because such values are strongly dependent on formulation composition, dissolution medium, geometry, agitation and the criterion used to define release; a single representative figure detached from its assay context is not a property of the chemistry and should not be read as one (Section 2.3). This review is narrative rather than systematic: no record-level screening counts are reported, the selection is not exhaustive, and the limitations that follow are stated in Section “Limitations of This Review”.

This review’s relationship with existing reviews is as follows: The subject matter of this review is well served by the recent secondary literature. There are comprehensive treatments of smart hydrogels [1,14], nanoparticle–hydrogel hybrid materials [11,15], nanomaterial-integrated hydrogel composites [12], stimuli-responsive nanocarriers, in general [4,6], and tumor-microenvironment-responsive polymer platforms, in particular [34], the translational record of nanomedicine [9,10] and machine-learning-assisted formulations [35,36]. Table 1 sets out what each of these covers and where the present review differs. We do not claim broader coverage than these works; several are deeper than this one within their own domain.

**Figure 2:**
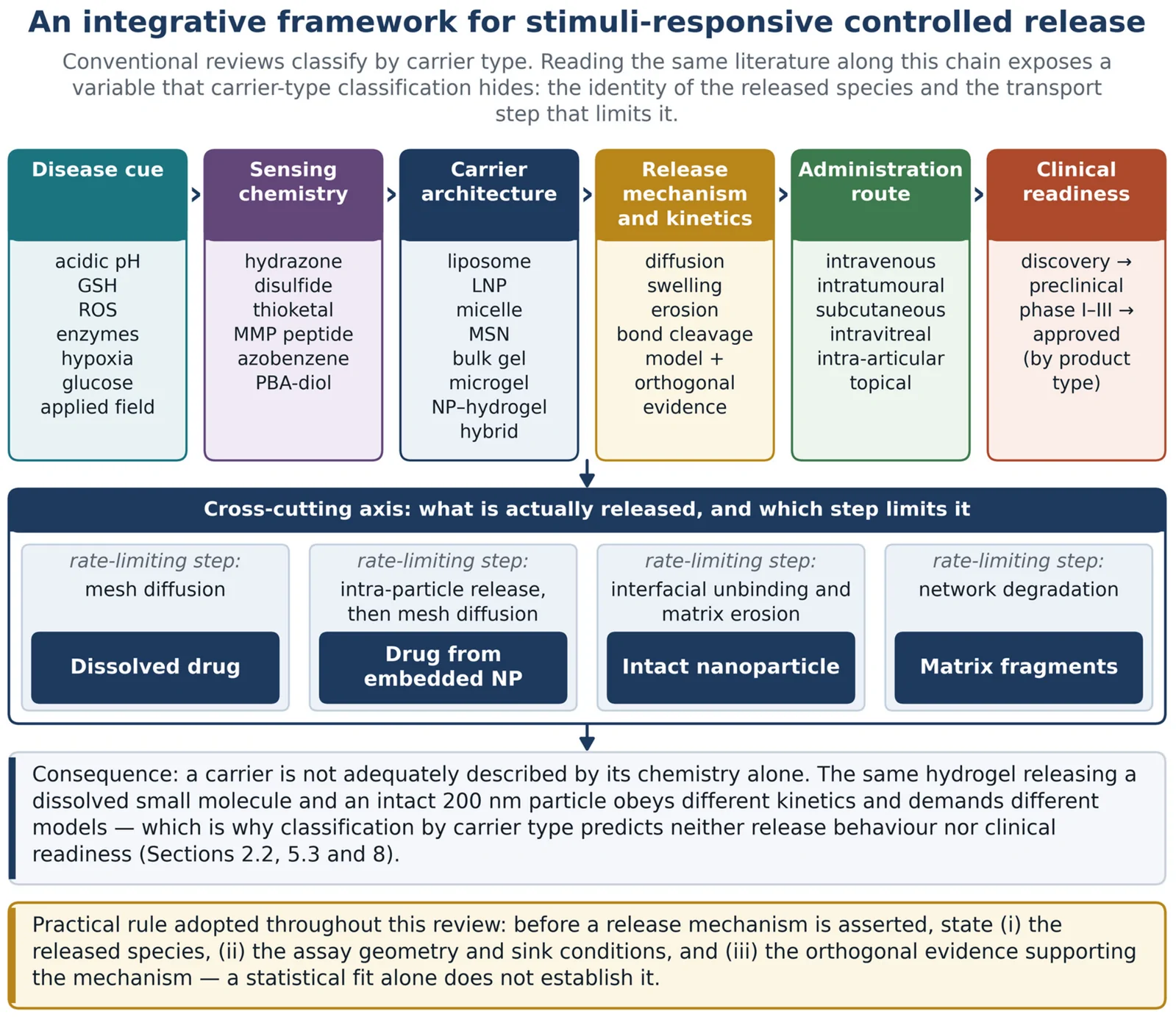
The integrative framework used throughout this review. Reading the literature along the chain from disease cue to clinical readiness exposes the released species and the rate-limiting transport step as the variable that carrier-type classification does not encode. Original figure created by the authors using Matplotlib 3.10.8 (Python 3.12.3).

This review adds the following: The contribution is not additional coverage but a different reading of the same literature. Reviews in this field, including those in Table 1, are almost universally organized by carrier type: one chapter on liposomes, one on polymeric particles, one on hydrogels, and one on hybrids. That organization is natural for a materials survey, but it conceals something. Here, the literature is instead read along the chain set out in Figure 2—disease cue, sensing chemistry, carrier architecture, release mechanism and kinetics, administration route, and clinical readiness—and read that way, it exposes a single cross-cutting variable: the identity of the species that is actually released, and the transport step that limits it. Four species are possible (dissolved drug, drug liberated from an embedded nanoparticle, an intact nanoparticle, and matrix fragments). They obey different kinetics, and they are not distinguished by the carrier-type classification.

Three consequences follow, and they are developed in Section 2.3, Section 5.3, Section 5.4 and Section 8 rather than asserted here. The power-law exponent analysis that describes dissolved-drug release from swellable matrices fits intact-particle escape from a nanocomposite hydrogel markedly worse than a first-order expression with a burst term, so an exponent transplanted across the two cases is interpreted mechanistically when it should not be [16,29]. A statistical fit does not by itself identify a mechanism, and because apparent release kinetics depend strongly on the release assay itself—dialysis membranes can mask burst release through slow permeation, membrane saturation and unstirred layers—mechanistic claims require orthogonal evidence and full reporting of assay conditions [37,38,39,40]. And when the same literature is examined against clinical readiness, the carriers that have reached patients are grouped not by sophistication of stimulus chemistry but by administration route and regulatory product type, which is the pattern Figure 8 is constructed to make visible. Demonstrating any of this requires the dissolved-drug baseline that only the standalone platforms of Section 3 and Section 4 provide; it could not be shown from the hybrid literature alone. The breadth of this review is, therefore, a condition of its argument rather than a substitute for depth.

## 2. Fundamentals of Sustained and Stimuli-Responsive Release

Designing a vehicle that delivers an API to its biological target with the right amount, in the right place, and at the right time requires that the formulator first understands the physical, chemical and biological forces that govern drug egress from a carrier. This section outlines those forces in three steps: the elementary release mechanisms operating in nanoparticulate and hydrogel matrices (Section 2.2), the mathematical models used to describe and predict them (Section 2.3), and the classification of stimuli that smart carriers exploit to trigger or modulate release on demand (Section 2.4).

### 2.1. Definitions, Conceptual Boundaries and Inclusion Criteria

Terms in this field are used loosely and often interchangeably, which makes claims difficult to compare across studies. Before the mechanisms are examined, the vocabulary used throughout this review is, therefore, fixed explicitly (Table 2), and the inclusion criteria of Section 1 are aligned to it.

Two boundary cases deserve comment because they are frequently, and, in our view, incorrectly, presented as controlled release. Active targeting—the decoration of a carrier with a ligand for a receptor overexpressed on diseased cells—alters where a carrier accumulates and how it is internalized. It does not by itself alter when or at what rate cargo leaves the carrier, and a targeted carrier with an uncontrolled release profile delivers its payload no more controllably than an untargeted one. Similarly, externally assisted delivery such as MR-guided focused ultrasound opening of the blood–brain barrier modifies a biological barrier rather than the carrier and permits a co-administered agent to enter a compartment it would otherwise be excluded from [42]. This is a genuine and clinically important capability, but it is barrier modulation, not controlled cargo release, and conflating the two inflates the apparent clinical maturity of stimuli-responsive delivery. Where such systems appear in this review—including in Section 7.4 and in Figure 8—they are labeled as barrier modulation or as drug–device combinations accordingly. Systems in which an exogenous field both opens a barrier and triggers cargo release from the carrier belong to both categories and are identified as such.

### 2.2. Release Mechanisms

For both nanoparticulate and hydrogel vehicles, drug release is rarely the consequence of a single, isolated process; it is almost always the convolution of several elementary mechanisms acting in parallel or in sequence [27,43]. Diffusion of the dissolved API through a continuous polymer matrix or through the hydrated mesh of a hydrogel is the dominant mechanism for non-degradable, non-swelling carriers and is described by Fick’s first and second laws. When the characteristic time of polymer chain relaxation (τr) is much longer than that of solvent penetration (τs), release is purely Fickian; when τr ≈ τs, the two processes couple and produce anomalous (non-Fickian) transport, in which the apparent diffusion coefficient becomes time- or position-dependent [27,43]. In the limiting case where polymer relaxation is rate-limiting, a sharp moving solvent front advances at constant velocity and the system shows Case II transport, characterized by near-zero-order release kinetics that are particularly attractive for sustained delivery [43,44].

Beyond Case II lies a further regime, Super Case II transport, in which release is faster than the constant-velocity front of Case II and the diffusional exponent exceeds unity for a slab (n > 1, with correspondingly higher thresholds for cylindrical and spherical geometries). It arises when the advancing solvent front accelerates rather than propagating at constant velocity, typically because swelling generates internal stresses that crack or delaminate the glassy core because plasticization progressively lowers the local glass transition temperature ahead of the front, or because matrix erosion begins to contribute while relaxation is still rate-limiting [27,43,44]. Super Case II is reported in highly swellable hydrophilic matrices and in some nanogels, and it matters practically because it is the one regime in which the release rate rises with time, producing a late-phase acceleration that is undesirable in a sustained-release product and easily mistaken for dose dumping.

These transport regimes describe how a dissolved molecule moves through a network whose structure is treated as fixed. In most hydrogels and in hydrophilic glassy matrices, the network is not fixed: it takes up solvent and reorganizes as release proceeds, and that reorganization becomes the controlling process.

Swelling-controlled release dominates in hydrogels and in glassy hydrophilic polymeric matrices. Solvent ingress lowers the local glass transition temperature, generates a rubbery gel layer at the matrix surface, and progressively widens the diffusional pathway, allowing the entrapped API to diffuse outward; the mesh size of the swollen network and the radius of gyration of the drug together determine whether release is hindered, free or geometrically obstructed [11,12,43]. Erosion-controlled release operates in biodegradable polymeric carriers such as poly(lactic-co-glycolic acid) (PLGA), polyanhydrides and aliphatic polyesters. Surface erosion produces near-zero-order kinetics because the drug-loaded volume shrinks at a constant rate, whereas bulk erosion—typical of PLGA microspheres—produces a characteristic triphasic profile (an initial burst, lag and accelerated final phase) reflecting the autocatalytic acid-catalyzed hydrolysis of internal ester bonds [45,46]. Dissolution and partitioning at the carrier–medium interface, osmotic pumping in semipermeable systems, and stimuli-triggered cleavage of labile linkers (acid-labile hydrazones and acetals; reducible disulfides; ROS-cleavable thioketals; and enzyme-cleavable peptide sequences) further enrich the kinetic palette available to the formulator [5,6,19,47].

### 2.3. Mathematical Modeling of Release

Because each of these mechanisms imprints a distinct mathematical signature on the cumulative release curve, kinetic modeling has become a routine—and, when properly applied, mechanistically informative—step in formulation development [27,29]. The most widely used empirical and semi-empirical models are summarized in Table 3.

Zero-order kinetics (M_t_/M_∞_ = k_0_·t) describe a constant release rate independent of remaining drug amount; they are the therapeutic ideal for chronic dosing and are approached experimentally by reservoir devices, surface-eroding matrices and well-designed osmotic pumps [27,28,44]. First-order kinetics (M_t_/M_∞_ = 1 − exp(−k_1_·t)) represent release that is proportional to the remaining payload and are typical of dissolution-limited systems and liposomal release in sink conditions [27]. Higuchi’s model (M_t_/M_∞_ = kH·t^1/2^) describes Fickian diffusion from a homogeneous, non-swelling planar matrix and remains the benchmark for ointments, transdermal patches and matrix tablets [27,29]. The Hixson–Crowell cube root law applies when the geometric surface area of dissolving particles changes during release [27,29].

The most versatile and widely applied empirical descriptor is the Korsmeyer–Peppas (Ritger–Peppas) power law, M_t_/M_∞_ = kKP·t^n^, where the diffusional exponent n discriminates between transport mechanisms in geometry-specific ways: for a thin slab, *n* ≈ 0.5 corresponds to Fickian diffusion and *n* = 1.0 to Case II transport, with intermediate values denoting anomalous transport; for cylinders, the Fickian limit is *n* ≈ 0.45, and for spheres, *n* ≈ 0.43 [27,29,47]. The Peppas–Sahlin model splits release into additive Fickian and relaxational contributions (M_t_/M_∞_ = k1·t^m^ + k2·t^2m^), enabling the designer to quantify the relative weight of each [27,48]. The Weibull function (M_t_/M_∞_ = 1 − exp[−(t/τ)^β^]) is an empirical but flexible distribution function that often fits experimental release data better than any of the mechanistic models, although its physical interpretation is debated; recent work using fractional calculus and Mittag-Leffler functions provides an alternative mechanistic foundation [27,28].

A comparative analysis of six classical models on heterogeneous nanocarrier release datasets concluded that the Korsmeyer–Peppas equation provides the best statistical fit (lowest χ^2^ per degree of freedom) across the broadest range of nanoparticulate and hydrogel systems [29], a finding reproduced in recent studies of PLGA depots [46], though not universally: in nanocomposite systems releasing intact nanoparticles rather than dissolved drug, the Korsmeyer–Peppas power law can fit markedly worse than first-order descriptions (Section 5.3) [16]. Importantly, however, several authors caution that mechanistic conclusions drawn from n alone—particularly for swelling, eroding or multi-component systems—can be misleading; orthogonal evidence (differential scanning calorimetry (DSC), gravimetric swelling, and microscopy of the eroding front) should accompany kinetic fitting wherever possible [43].

Over the past two years, classical kinetic modeling has been increasingly complemented by machine learning (ML) approaches. Random forests, gradient-boosted regressors, support vector machines and deep neural networks are now used to predict early-phase, late-phase and complete release profiles from formulation descriptors (polymer molecular weight, copolymer ratio, drug load, particle size, zeta potential, and dissolution medium composition) [35,36,49]. A 2024 perspective in the *Journal of Controlled Release* by Gormley argued that ML methods will become indispensable in mapping the multi-dimensional formulation spaces typical of long-acting injectables and nanocomposite hydrogels [50], and a 2023 study in *Nature Communications* trained eleven algorithms on 181 in vitro release profiles spanning 43 unique drug–polymer combinations and identified a light-gradient-boosting model as the most accurate predictor of fractional release on held-out drug–polymer splits. Shapley additive explanation analysis of that model ranked elapsed time, fractional release at 24 h, drug molecular weight and polymer molecular weight as the dominant contributors, with the remaining formulation descriptors—including the lactide-to-glycolide ratio—contributing comparatively little [49]. Recent reviews have documented the same approach applied to liposome design and lipid nanoparticle optimization [35,36], and a retrospective analysis of over 900 three-dimensionally printed drug delivery systems extended it to printed dosage forms [51]. The current bottleneck is widely recognized to be data quality: open-access, well-annotated in vitro release databases, harmonized release-testing methodologies, and FAIR (findable, accessible, interoperable, reusable) reporting standards are being actively built to unlock the full power of these approaches [52].

What a kinetic fit does and does not establish is detailed as follows. The models in Table 3 are routinely used to infer a mechanism from the shape of a release curve, and this inference is weaker than the literature generally acknowledges. Each model is derived under assumptions—about geometry, boundary conditions, the constancy of the diffusion coefficient, the maintenance of sink conditions and the fraction of the curve over which the expression is valid—that are frequently violated by the systems to which it is applied. Table 4 sets out these assumptions and validity domains explicitly. Three specific cautions follow.

First, the models are not mutually exclusive descriptions of distinct physics, and several will often fit the same dataset to within its experimental error. Discriminating between them requires an information criterion that penalizes parameter count (the Akaike or Bayesian information criterion) rather than the coefficient of determination alone, which cannot decrease when parameters are added; it requires inspection of the residuals for structure, since systematic curvature in the residuals indicates model misspecification even when R^2^ is high; and it requires the fitting range to be stated, because Higuchi’s and the Korsmeyer–Peppas expressions are formally valid only over the initial portion of the curve, conventionally the first 60% and 60–70% of cumulative release respectively. A power-law exponent extracted from a full profile is not the quantity the model defines. Second, the diffusional exponent is geometry-specific, so the Fickian and Case II boundaries differ for slabs, cylinders and spheres (Table 3), and reporting n without stating the geometry renders it uninterpretable.

Third, and most consequentially, the apparent release kinetics depend on the release assay as much as on the formulation. There is no compendial standard for release testing of colloidal carriers, and the principal methods—sample-and-separate, dialysis membrane and continuous-flow techniques—do not return equivalent profiles from the same material [39,40,53]. Dialysis is the most widely used and the most artefact-prone: because the measured signal convolves release from the carrier with permeation of free drug across the membrane, a rapid burst can be masked entirely by slow membrane transport, by temporary saturation of the membrane, and by the unstirred layer inside the donor compartment [38]. These are not marginal effects. In a dialysis-based system, the membrane permeation rate constant more than doubled between low and high stirring rates [54]; in a direct comparison of three methods applied to a single mesoporous silica formulation, the profile ranged from a burst-and-plateau to sustained release approaching 60% over 48 h purely as a function of method and agitation [37]; and in PLGA nanocapsules, a sample-and-separate approach resolved a burst phase that the dialysis comparator did not detect [38]. Comparative studies of PLGA nanoparticles and of solid lipid microparticles reach the same conclusion from different materials [55]. Sink conditions, the membrane molecular weight cut-off, the agitation mode and rate, and the permeability of the membrane measured before and after the experiment should, therefore, be reported alongside every release dataset [40], and the release response for dialysis-based methods should be expressed with the membrane permeation rate constant determined independently.

The practical consequence adopted throughout this review is that a statistical fit is treated as a description of the data, not as evidence of a mechanism. A mechanistic claim is accepted here only where the kinetic analysis is accompanied by orthogonal evidence bearing on the process invoked: gravimetric or imaging measurement of swelling for a relaxation-controlled claim, mass loss or molecular weight decline for erosion, differential scanning calorimetry (DSC) or spectroscopic confirmation of the physical state of the entrapped drug, microscopy of the eroding front, or direct chemical demonstration of linker cleavage [43]. Where a claim in the primary literature rests on curve fitting alone, it is reported here as an observed profile rather than as an identified mechanism.

### 2.4. Classification of Stimuli

A defining feature of contemporary delivery vehicles is the deliberate exploitation of stimuli to trigger or modulate cargo release in space and time. Stimuli are conventionally divided into endogenous (internal) cues—pathological deviations from healthy physiology—and exogenous (external) cues applied by the operator [4,5,6,34]. Endogenous stimuli include the mildly acidic extracellular pH of solid tumors (~6.5–7.2), the more acidic environment of endosomes (5.5–6.8) and lysosomes (4.5–5.5); intracellular glutathione (GSH) concentrations 100–1000-fold higher in tumor cells (~2–10 mM) than in plasma (~2 µM); elevated reactive oxygen species (ROS) levels in inflamed and malignant tissue; overexpression of matrix metalloproteinases, hyaluronidases, cathepsins and esterases in tumor stroma and infected wounds; chronic hypoxia in poorly vascularized tumor cores (pO_2_ < 10 mmHg); hyperglycemia in diabetes; and elevated ATP in cancer cells [5,6,8,19,34,47,56]. Exogenous stimuli include local mild hyperthermia (40–43 °C); near-infrared, visible and UV light; alternating magnetic fields; focused ultrasound; and applied electric fields [6,22,23,24,34].

Each class has characteristic strengths and limitations. Endogenous triggers are inherently disease-targeted, requiring no operator intervention, but their magnitude and spatial heterogeneity vary widely between patients and tumor regions, making consistent activation difficult [5,34,56]. Exogenous triggers offer precise spatiotemporal control and are easily integrated with imaging, but their tissue penetration (limited for UV and visible light), focal precision (limited for systemic heating) and equipment requirements (focused ultrasound transducers and MRI-coupled magnetic actuators) constrain clinical implementation [6,22,23]. The contemporary trend, examined in detail in Section 6, is, therefore, towards multi-stimuli or AND-gated systems that combine an endogenous and an exogenous cue (e.g., pH plus NIR; redox plus ultrasound), thereby reducing off-target activation while retaining operator control [25,26,34]. A recent 2025 *Angewandte Chemie* review of tumor-microenvironment-responsive polymer platforms noted that more than half of newly reported anticancer carriers in 2023–2024 were dual- or triple-responsive [34].

### 2.5. An Integrative Framework: The Released Species as the Organizing Variable

The elements assembled so far—transport regimes, kinetic models and their limits, and the catalog of stimuli—are conventionally deployed carrier by carrier. Figure 2 sets out the alternative organization used in this review. Reading a delivery system as a chain from the pathological cue that it senses, through the chemistry that transduces that cue, the architecture that carries the cargo, the mechanism and kinetics by which the cargo leaves, the route by which the construct is administered, and finally, the stage of clinical development it has reached, has two advantages. It keeps the biological problem rather than the material in the foreground, and it makes visible a variable that carrier-type classification does not encode.

That variable is the released species. A carrier does not simply ‘release drug’: depending on its architecture, it may release dissolved drug through the network mesh, drug liberated from an embedded nanoparticle after that particle has been reached by the medium, an intact drug-loaded nanoparticle that will liberate its cargo elsewhere, or fragments of a degrading matrix carrying adsorbed cargo. Each corresponds to a different rate-limiting transport step—mesh diffusion, intra-particle release, interfacial unbinding and matrix erosion, or network degradation—and, therefore, to a different kinetic signature and a different set of models that may legitimately be applied. The same hydrogel, loaded with a small molecule in one case and with drug-loaded 200 nm particles in the other, is two different release systems that happen to share a matrix.

This has an immediate methodological corollary and a slower-acting translational one. The methodological corollary is that release data should identify the species measured: an assay that quantifies drug in the receiving medium does not distinguish free drug from drug still inside an escaped particle, and the distinction changes both the kinetics and the biological consequence. The translational corollary is developed in Section 8: when carriers are ordered by clinical readiness rather than by chemistry, the successful ones cluster by administration route and regulatory product type, not by the elegance of their trigger chemistry. Both are visible only when the standalone and hybrid literatures are read against one another, which is the reason this review retains all three material classes.

## 3. Nanoparticulate Vehicles

**Figure 3:**
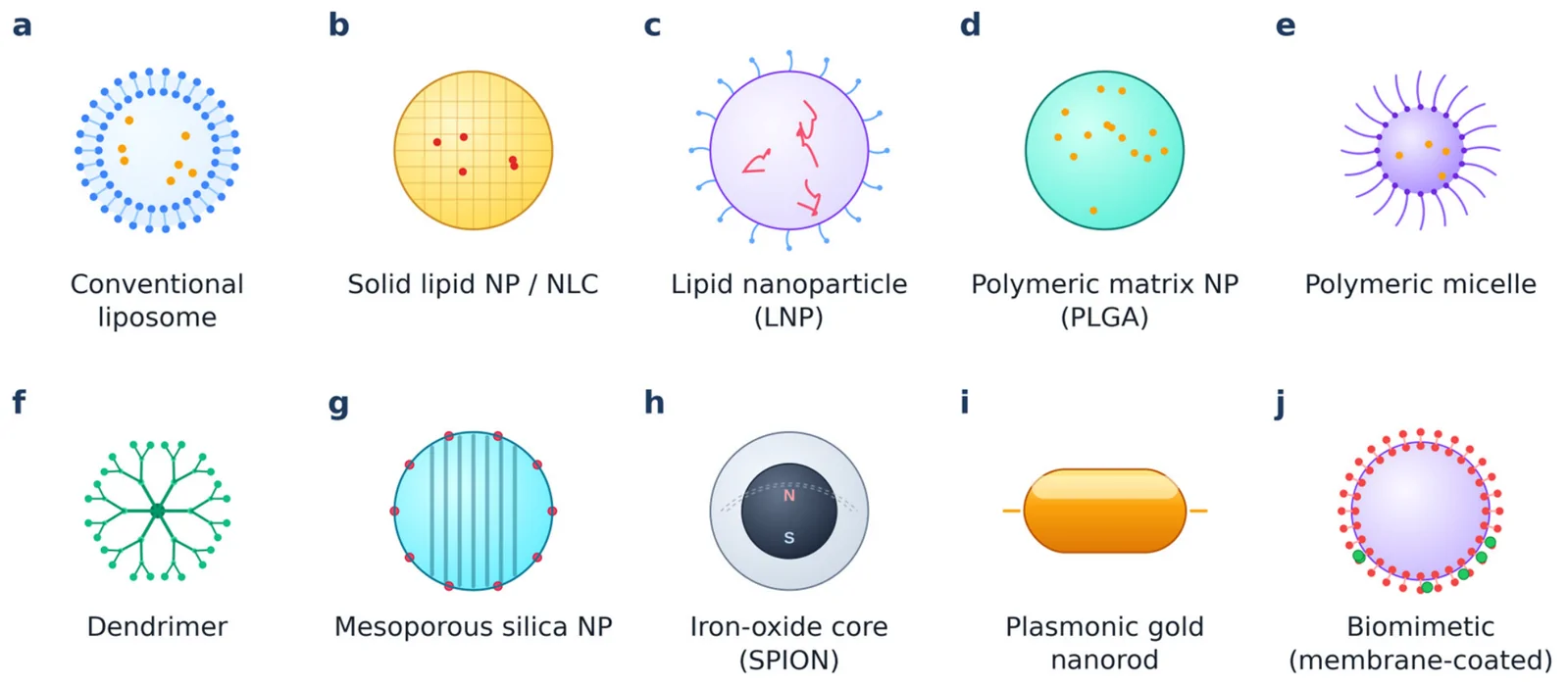
Schematic comparison of the principal nanoparticulate architectures used for sustained and stimuli-responsive controlled release. (**a**) Conventional liposome: phospholipid bilayer enclosing an aqueous core; (**b**) solid lipid nanoparticle/nanostructured lipid carrier: solid (or imperfect solid–liquid) lipid matrix; (**c**) lipid nanoparticle: ionizable lipid + helper lipids + cholesterol + PEG–lipid encapsulating nucleic acid; (**d**) polymeric matrix nanoparticle (e.g., PLGA) with drug dispersed in the polymer matrix; (**e**) polymeric micelle: amphiphilic block–copolymer core–shell self-assembly; (**f**) dendrimer: hyperbranched, generation-defined macromolecule; (**g**) mesoporous silica nanoparticle with surface-grafted “nanovalves”; (**h**) iron-oxide superparamagnetic core with polymer shell; (**i**) plasmonic gold nanorod; and (**j**) cell-membrane-coated biomimetic nanoparticle. Original figure created by the authors using Matplotlib 3.10.8 (Python 3.12.3).

The contemporary toolkit of nanoparticulate carriers spans four broad families—lipid-based, polymeric, inorganic/hybrid, and biomimetic—each with characteristic architectures, encapsulation chemistries, release behaviors and translational maturity (Figure 3; Table 5). The choice between them is rarely arbitrary: it is dictated by the API’s molecular class (small molecule vs. peptide, protein, or oligonucleotide), the intended administration route, the desired release kinetics, and the regulatory pathway envisaged.

### 3.1. Lipid-Based Nanoparticles

Lipid-based carriers remain the most clinically advanced nanoparticulate platform. Conventional liposomes—phospholipid bilayer vesicles enclosing an aqueous core—were the first nanomedicines to reach the market (Doxil^®^, 1995) and continue to underpin approved formulations of doxorubicin, daunorubicin, irinotecan, vincristine and amphotericin B [7,9]. Their bilayer structure permits dual encapsulation of hydrophilic drugs in the lumen and lipophilic drugs in the lipid leaflet, while PEGylation of the outer surface (“stealth” liposomes) extends circulation by minimizing opsonization [7,26]. Solid lipid nanoparticles (SLNs) and second-generation nanostructured lipid carriers (NLCs) replace the bilayer with a solid lipid matrix (SLN) or an intentionally imperfect blend of solid and liquid lipids (NLC), the latter providing higher drug loading, reduced expulsion during storage, and modulated release profiles [57,58,73]. Both are produced by high-pressure homogenization or solvent injection from physiological, GRAS-listed lipids and have proven particularly suitable for ocular, dermal and oral delivery of poorly soluble drugs [58,73]. Lipid nanoparticles (LNPs), in the modern sense—composed of an ionizable cationic lipid, a structural phospholipid, cholesterol and a PEG–lipid—have come to dominate nucleic acid delivery, enabling siRNA (Onpattro^®^, 2018) and mRNA (Comirnaty^®^ and Spikevax^®^ COVID-19 vaccines) therapeutics [7,10,59]. Their pH-dependent ionization permits efficient cytosolic release after endosomal acidification, and a recent translational review documented dozens of LNP-based formulations in clinical evaluation across oncology, infectious diseases and rare genetic disorders [7,10].

### 3.2. Polymeric Nanoparticles

Polymeric nanoparticles encompass solid matrices, core–shell self-assemblies and hyperbranched architectures. PLGA and other aliphatic polyesters (PLA and PCL) remain the polymeric workhorse, owing to FDA/EMA approval, a tunable lactide:glycolide ratio (which dictates the degradation rate from weeks to months), compatibility with small molecules, peptides and nucleic acids, and well-characterized triphasic release behavior [45,46,60]. Chitosan, alginate and other polysaccharide nanoparticles offer mucoadhesion, intrinsic biocompatibility and pH-sensitive ionization, which is exploited in oral and intranasal delivery [60]. Polymeric micelles, formed by self-assembly of amphiphilic block copolymers (most commonly PEG-b-PLA, PEG-b-PCL, and PEG-b-poly(amino acid)s) in aqueous solution, are unparalleled solubilizers for highly hydrophobic APIs such as paclitaxel and docetaxel, with several formulations (Genexol-PM^®^, Nanoxel^®^, and NK105) already approved or in late-stage trials in Asia [61,62,63]. Their core–shell architecture enables stimuli-responsive disassembly (pH, redox, or ROS) and on-demand release, and a 2024 review summarized the emerging field of TME-responsive micelles for precision oncology [63,74]. The clinical bottleneck for micelles remains kinetic instability upon dilution below the critical micelle concentration (CMC), addressed by core-crosslinking, mixed-micelle strategies and PEG-assisted assembly [62].

Dendrimers—perfectly branched, monodisperse, generation-defined macromolecules with well-defined surface group densities—occupy a unique position between small molecules and polymeric nanoparticles. Polyamidoamine (PAMAM), poly(propylene imine) (PPI) and polylysine dendrimers can encapsulate guests in internal cavities or conjugate them to surface termini, giving precise control over stoichiometry [64,65]. Recent work has focused on engineering surface chemistry to mitigate the cytotoxicity associated with cationic terminal amines while retaining cellular uptake, on dendrimer–drug conjugates with cleavable linkers, and on dendrimer-mediated dermal, ocular and cerebral delivery [64,65,75]. Hyperbranched and hybrid dendritic–polyester systems are now extending these concepts toward gram-scale, GMP-compatible manufacture.

Hyperbranched polymers occupy the middle ground between the perfectly defined dendrimer and the statistically branched polymer, and they matter here for a translational reason. Where dendrimers are built generation by generation through iterative protection and deprotection—monodisperse but expensive and difficult to scale—and hyperbranched polymers are obtained in a single step from AB_2_, A_2_ + B_3_ or self-condensing vinyl polymerization, at the cost of a distribution of molecular weights and an incomplete degree of branching, typically 0.2–0.5, rather than the unity of an ideal dendrimer [76,77]. Their globular architecture, nevertheless, retains the features that make dendritic carriers useful: intramolecular cavities for guest encapsulation, a high density of peripheral groups for conjugation, and low solution viscosity relative to a linear polymer of equivalent mass [76]. Recent one-pot routes—including latent inimer and photoswitchable catalytic strategies—have made the degree of branching itself a controllable variable rather than an uncontrolled outcome [77].

Two features are directly relevant to controlled release. Drug loading by covalent conjugation to the periphery reaches values unattainable by physical encapsulation: hyperbranched polydisulfide–camptothecin conjugates carry approximately 40% drug by mass and release the active drug through a glutathione-triggered cascade in which disulfide cleavage is followed by intramolecular attack on the neighboring carbonate, with the branched conjugate substantially outperforming its linear analog in intracellular delivery [78]. More importantly, a hyperbranched prodrug can function as a unimolecular micelle: because the amphiphilic architecture is contained within a single macromolecule, it does not disassemble on dilution below a critical micelle concentration. A redox-responsive doxorubicin–hyperbranched conjugate of 62% drug content and 122 nm hydrodynamic diameter released 60.9% of its payload over 85 h under glutathione challenge, while premature leakage remained at 3.2% [79]. This directly addresses the kinetic instability upon dilution that remains the principal translational weakness of multimolecular polymeric micelles (Section 3.2 above), and it does so architecturally rather than by crosslinking the core. The same architecture is used outside oncology: a hyperbranched polylysine bearing oxidizable phenylthio groups self-assembles with selenomethionine into a reactive oxygen species-responsive carrier for abdominal aortic aneurysm, as discussed in Section 7.5.

### 3.3. Inorganic and Hybrid Nanoparticles

Inorganic platforms exploit physical properties (porosity, magnetism, and plasmonic absorption) that organic carriers cannot replicate. Mesoporous silica nanoparticles (MSNs) combine very high specific surface areas (~800–1000 m^2^/g), tunable pore diameters (2–10 nm), straightforward functionalization through abundant surface silanols, and the possibility of installing molecular “nanovalves” at pore mouths to gate release in response to pH, redox, light or enzymes [19,20,80]. Hybrid MSNs incorporating polymer shells, lipid bilayers, gold nanoshells or magnetic cores generated multifunctional theranostic platforms over the 2021–2025 period [19,20]. Gold nanoparticles—spheres, rods, cages and stars—offer plasmonic absorption that can be tuned across the visible-to-NIR window for photothermal therapy and photo-triggered drug release, while also serving as facile scaffolds for thiolated drug–DNA conjugates [6,22]. Iron oxide nanoparticles (IONPs/SPIONs), FDA-approved as MRI contrast agents (e.g., Feraheme^®^), enable magnetically guided accumulation, alternating-magnetic-field hyperthermia (~42–45 °C) and stimuli-coupled release when embedded in thermo- or pH-responsive shells [23,66]. Metal–organic frameworks (MOFs) are crystalline coordination polymers built from metal nodes and organic linkers, offering Brunauer–Emmett–Teller surface areas up to 6000 m^2^/g and unprecedented drug-loading capacities (up to 60% *w*/*w* for some Fe^3+^–carboxylate MIL-type frameworks). Recent reviews documented over 400 MOF–drug delivery papers in 2024 alone, with stimuli-responsive Zr-, Zn-, Fe- and Cu-based MOFs reaching preclinical efficacy in oncology and immunotherapy [67,68,69]. Carbon-based platforms (carbon dots, graphene oxide, and carbon nanotubes) and upconverting nanoparticles (UCNPs) complete this family, the latter enabling NIR-to-UV light conversion that overcomes the tissue penetration limit of UV photo-triggers [6,22].

Despite the breadth of this toolkit, a sober assessment is warranted. A widely cited 2016 meta-analysis by Wilhelm et al. of pre-clinical tumor studies concluded that the median fraction of an injected nanoparticle dose reaching a solid tumor is approximately 0.7% [81]. That figure has been disputed in detail—alternative analyses incorporating active-targeting and post-2016 data put the median closer to 1–2% [41,82]—but the qualitative finding has held: the EPR effect alone is rarely sufficient for clinically meaningful tumor accumulation in patients, and active-targeting moieties improve delivery only modestly when integrated over heterogeneous tumor populations [9,10,41,83]. This is the central translational tension of the field, and it informs the renewed interest in locoregional administration via hydrogels (Section 4), hybrid NP–hydrogel depots (Section 5), stimuli-responsive systems with sharp on/off contrast (Section 6), and patient-stratified clinical trial designs (Section 8).

### 3.4. Biomimetic and Cell-Membrane-Coated Nanoparticles

A more recent—and still rapidly evolving—strategy is to coat synthetic cores (most often PLGA, mesoporous silica or magnetic NPs) with intact plasma membranes harvested from red blood cells, platelets, leukocytes, mesenchymal stem cells, cancer cells or bacterial extracellular vesicles, so that the resulting “cloaked” particles inherit the source cell’s surface antigens, immune-evasion markers (CD47 “don’t eat me” signals) and homotypic targeting [70,71,72]. RBC-coated cores show prolonged circulation; platelet-coated particles target injured vasculature and circulating tumor cells; cancer-cell-membrane-coated particles exploit homotypic adhesion to seek their cell of origin; and macrophage-coated particles accumulate at sites of inflammation [70,71]. Recent 2024–2025 work extends the concept to membrane-cloaked MOFs and to genetically engineered membranes that present designer ligands [71,72]. Translational obstacles—batch-to-batch membrane variability, scalable extrusion, sterilization compatibility, and regulatory classification of a hybrid biological–synthetic product—are now the focus of intensive engineering effort [72].

A 2024 synthesis of biomimetic delivery systems placed these approaches in a wider frame, treating erythrocyte, platelet, leukocyte, and stem-cell and cancer-cell membrane coatings alongside exosome-based and virus-mimetic carriers as a single design strategy whose common principle is the transfer of a biological interface onto a synthetic core, and identifying interface reproducibility rather than core chemistry as the limiting variable for translation [84].

## 4. Hydrogel Vehicles

**Figure 4:**
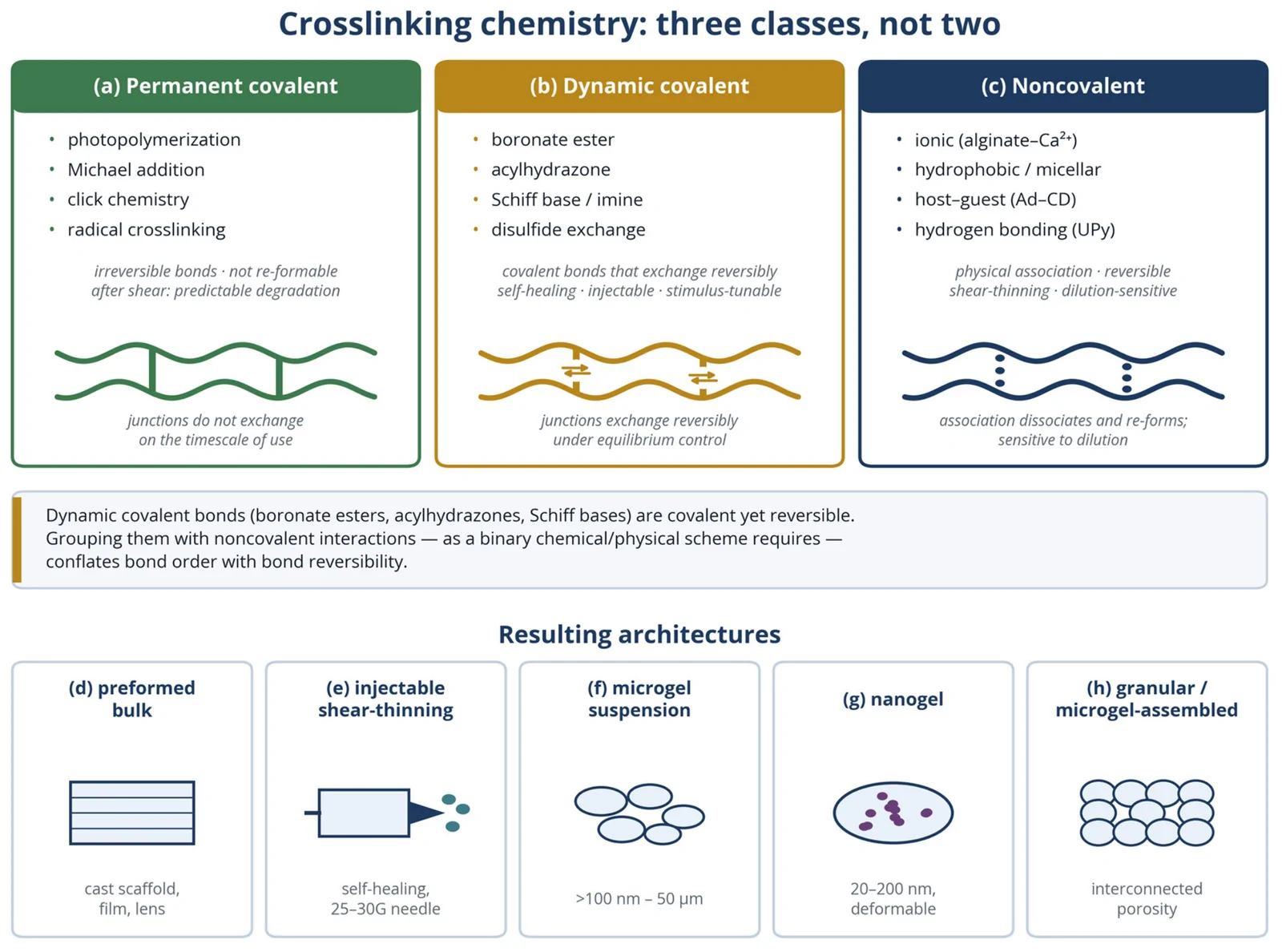
Crosslinking chemistry and architectures of hydrogel vehicles. (Top) Three classes of junction, distinguished by bond order and reversibility rather than by a binary chemical/physical scheme. (**a**) Permanent covalent: photopolymerization, Michael addition, and click chemistry; (**b**) dynamic covalent: boronate ester, acylhydrazone, Schiff base, disulfide exchange, and covalent yet reversible; and (**c**) noncovalent: ionic, hydrophobic, host–guest, and hydrogen bonding. (Bottom) Resulting architectures: (**d**) preformed bulk; (**e**) injectable shear-thinning and self-healing; (**f**) microgel suspension; (**g**) nanogel; and (**h**) granular, microgel-assembled with interconnected porosity. Original figure created by the authors using Matplotlib 3.10.8 (Python 3.12.3).

Where nanoparticles excel at navigating the bloodstream, hydrogels excel at staying where they are placed. Their three-dimensional, hydrophilic polymer network—typically containing 70–99% water—combines macroscopic mechanical compliance (storage moduli of 100 Pa to >100 kPa) with mesh sizes (1–100 nm) that determine which solutes can diffuse and at what rate [11,12,85]. For sustained controlled release, this anatomy translates into four practical advantages: large drug-loading reservoirs; soft, ECM-mimetic mechanics that minimize foreign-body response; permeability that can be tuned over orders of magnitude; and the option of delivering the carrier itself by syringe rather than by surgery [12,13,86]. Section 4 surveys the principal hydrogel architectures (Figure 4; Table 6), their crosslinking strategies, microgel/nanogel descendants, and the administration routes that have come to define the clinical landscape of hydrogel-based delivery.

### 4.1. Classification: Crosslinking Chemistry, Polymer Source and Administration Form

Hydrogels are classified along three orthogonal axes. The first, and the one most often misstated, is crosslinking chemistry. It is conventional to describe networks as either chemical or physical, but that binary conflates two independent properties—the order of the bond and its reversibility—and it forces dynamic covalent chemistries into a category where they do not belong. Three classes should be distinguished (Figure 4). Permanent covalent networks are formed by free-radical polymerization, photopolymerization, Michael addition or click chemistry; their junctions do not exchange on the timescale of use, which confers mechanical durability and predictable degradation but precludes post-formation injection and may leave residual reactive species [85,86,89]. Dynamic covalent networks—boronate esters, acylhydrazones, imines and Schiff bases, and thiol–disulfide exchange—are held together by genuine covalent bonds that, nevertheless, form and break reversibly under equilibrium control; they are, therefore, simultaneously covalent and self-healing, they are injectable, and their exchange rate is itself tunable by pH or by the presence of a competing diol, which is what makes boronate networks intrinsically glucose-responsive [12,85,96]. Noncovalent networks are assembled from ionic bridging (alginate–Ca^2+^), hydrophobic and micellar association, host–guest complexation, hydrogen bonding and stereocomplexation; they are the most readily reversible, the most injectable and the most sensitive to dilution [85,86,90,91]. Placing boronate esters and acylhydrazones among the physical crosslinks, as a two-class scheme requires, obscures precisely the property that makes them useful: they combine the bond strength of covalent chemistry with the reversibility of a physical association.

The practical consequence of this three-way distinction is that reversibility, not bond order, predicts the behaviors that matter for delivery—injectability, self-healing, shear-thinning and susceptibility to dilution—while bond order predicts mechanical persistence and degradation route. Noncovalent and dynamic covalent networks, therefore, share an application space that permanent covalent networks cannot enter, and the choice between them turns on the exchange kinetics required. A recent demonstration illustrates how far purely physical association can be taken: a thermosensitive poly(lactic acid-co-glycolic acid)–poly(ethylene glycol)–poly(lactic acid-co-glycolic acid) triblock copolymer that is a free-flowing sol at room temperature and forms a physically crosslinked gel at body temperature was used to support tracheal reconstruction, the weak physical junctions allowing cells to remodel the network in a way that a chemically crosslinked gel of comparable modulus does not permit [97]. The same reversibility that makes such a network cell-re-modellable also makes it erode, which is the mechanism by which it releases cargo.

By polymer source, hydrogels are natural—built from polysaccharides (alginate, chitosan, hyaluronic acid, dextran, and cellulose), proteins (collagen, gelatin, fibrin, silk, and elastin) or DNA—or synthetic, drawing from PEG, poly(vinyl alcohol), poly(acrylic acid), poly(N-isopropylacrylamide) (PNIPAM) and acrylamide derivatives [12,85,86]. Natural polymers offer biocompatibility, intrinsic bioactivity (cell adhesion motifs in gelatin, or RGD on fibrin) and biodegradability, but suffer from batch variability and sometimes immunogenic contaminants; synthetic polymers offer reproducibility, fine-tuned mechanics and well-defined chemistry, at the cost of inert biology [12,85,86,87]. Hybrid natural–synthetic networks (for example, gelatin methacryloyl (GelMA) with PEG-diacrylate) increasingly combine the best of both [13,86]. By administration form, hydrogels are either preformed bulk (cast or molded scaffolds, films, or lenses) or in situ-forming and injectable, administered as a fluid precursor that gels in response to body temperature (poloxamer 407 and methylcellulose), a physiological ion (alginate–Ca^2+^), pH (chitosan), enzymatic action (fibrin), photoirradiation (visible-light GelMA), or a self-mixing two-component formulation [11,12,86,89].

Quantitatively, drug release from a hydrogel matrix depends on the relative size of the diffusing solute and the network mesh. The Ogston obstruction model predicts the diffusivity ratio D/D_0_ = exp[−((r_s_ + r_n_)/ξ)^2^], where r_s_ is the solute hydrodynamic radius, r_n_ the polymer-fiber radius and ξ the mesh size; small molecules (r_s_ ≲ 1 nm), thus, diffuse essentially freely through most hydrogels, peptides and small proteins (1–3 nm) experience modest hindrance, and full-length immunoglobulins or virus-like particles (10–20 nm) are strongly hindered or sterically excluded [12,86]. Lustig and Peppas refined this picture with a screening-length-corrected expression that better matches macromolecular hydrogel data, and contemporary nanocomposite and granular hydrogels exploit this dependence precisely, tuning mesh size to release a small-molecule cargo over hours while retaining a co-encapsulated biologic for weeks [11,12,86]. When mesh sizes approach the drug radius, release becomes erosion-limited rather than diffusion-limited, and the mathematical models of Section 2.3 reach their applicability limits.

### 4.2. Self-Healing, Shear-Thinning, Supramolecular and Tough Hydrogels

The clinical move from open surgical placement to needle injection has driven intense interest in dynamic-crosslink hydrogels capable of shear-thinning flow under the high stress encountered in a syringe and self-healing restoration of mechanical integrity at the implantation site within seconds to minutes [11,85,89]. Supramolecular polymeric hydrogels—exemplified by host–guest networks such as adamantane–β-cyclodextrin (Ad–CD) and cucurbit [8] uril–azobenzene, ureido-pyrimidinone (UPy) quadruple hydrogen bonding, metal coordination chemistry, and polymer–nanoparticle (PNP) networks—provide such behavior without relying on covalent chemistry [11,85,89,90,91]. PNP hydrogels formed by hydrophobic association between dodecyl-modified hydroxypropyl methylcellulose (HPMC-C12) and PEG-PLA nanoparticles, developed by the Appel laboratory, have become a paradigmatic system: they are injectable through 30-gauge needles, encapsulate drugs ranging from small molecules to monoclonal antibodies and protein cytokines, and erode at controlled rates over weeks to months, with intravitreal, subcutaneous and intra-articular formulations now in advanced preclinical evaluation [11,33]. Liposomal hydrogels, in which liposomes themselves serve as multivalent crosslinkers for hydrophobically modified polymers, extend this concept to programmable orthogonal release of multiple protein drugs [11,98].

A 2023 *Chemical Reviews* article by Bertsch et al. consolidated the design rules for self-healing injectable hydrogels—emphasizing the trade-off between bond exchange kinetics, mechanical strength and cargo retention—and remains the most-cited contemporary reference for the field [99]. Complementing this dynamic-network paradigm, tough hydrogels—double networks (DNs), nanocomposite networks, slide-ring networks and ionically reinforced gels—combine soft, sacrificial primary networks with stiff secondary ones to reach moduli and toughness comparable to articular cartilage (~10^5^–10^6^ J/m^3^), enabling load-bearing implant applications and prolonged-residence wound dressings [86,92].

### 4.3. Microgels and Nanogels

Reducing hydrogel dimensions to the micrometer and nanometer scale produces microgels (>100 nm to >50 µm) and nanogels (typically 20–200 nm), which combine hydrogel-like swelling and stimuli responsiveness with the colloidal behavior of nanoparticles [30,93,94,95]. Synthesized by inverse-microemulsion polymerization, precipitation polymerization, microfluidic droplet templating or radiation-induced crosslinking, nanogels load both hydrophilic and hydrophobic APIs at high efficiency, achieve responsive volume-phase transitions to pH, temperature, redox and biomolecular cues, and, because they are deformable, can extravasate through tight endothelial junctions and traverse mucosal barriers more readily than rigid nanoparticles [30,93,94,95]. Recent reviews document the use of nanogels for vaccine delivery (antigen-loaded chitosan and mannose-decorated nanogels), brain delivery (RVG-modified PEG–polyamine nanogels crossing the blood–brain barrier), oral protein delivery (insulin nanogels), and stimuli-responsive cancer chemo-immunotherapy [30,94,95]. Microgels are increasingly being used as injectable building blocks of granular hydrogels—interconnected microgel assemblies whose interstitial porosity supports cell infiltration in situ, a property unattainable in conventional bulk hydrogels [11,17].

### 4.4. Routes of Administration

The form-factor versatility of hydrogels translates into a wide spectrum of administration routes. Injectable depots for subcutaneous, intramuscular, intratumoral, intra-articular and intravitreal delivery enable single-dose, weeks-to-months exposure for chemotherapeutics, biologics and gene therapies; many of the systems described above (PNP, liposomal hydrogels, supramolecular Ad–CD networks, in situ thermogelling poloxamers, and shear-thinning UPy hydrogels) are designed expressly for this route [11,33,89,98,100]. Oral and buccal hydrogels exploit mucoadhesion (chitosan and thiomers) and pH responsiveness to protect biologics from gastric acid, target the small intestine and bypass first-pass metabolism, with smart insulin-delivering hydrogels being a particularly active area in 2024–2025 [101,102]. Transdermal patches and microneedle hydrogels provide painless, controlled delivery across the stratum corneum [12]. Ocular hydrogels—preformed contact lenses, in situ-gelling eye drops, and intravitreal depots—extend residence time in a notoriously short-residence compartment [33,58]. Intranasal, vaginal, rectal and wound-bed applications complete the picture, the last being the most translationally advanced: multifunctional NP–hydrogel wound dressings combining antimicrobial silver or copper, pro-angiogenic factors, ROS-scavenging cerium oxide and glucose-responsive insulin release are entering clinical use for diabetic and chronic wounds [11,15,87].

### 4.5. Comparative Assessment: Matching Platform to Release Problem

The platforms in Table 6 are not interchangeable, and the choice between them is better made from the release requirement backwards than from the material forwards. Where the requirement is weeks to months of exposure to a biologic from a single subcutaneous or intravitreal administration, the binding constraint is erosion rate rather than mesh size, and polymer–nanoparticle and supramolecular networks are preferred because their erosion is governed by the unbinding kinetics of a defined association that can be engineered, whereas the degradation of a covalent network is governed by hydrolysis that cannot easily be decoupled from mechanics [11,33,98]. Where the requirement is a load-bearing residence in a mechanically demanding site, the constraint inverts: double-network and nanocomposite gels are the only class reaching cartilage-like toughness, and their release is correspondingly less tunable [86,92]. Where the requirement is cell infiltration—regenerative rather than purely pharmacological—granular microgel assemblies are effectively the only option, because interconnected macroporosity cannot be achieved in a bulk network without compromising it [11,17].

Two cross-cutting observations follow from comparing the platforms rather than describing them separately. First, the properties that make a hydrogel injectable—reversible junctions, shear-thinning, and rapid recovery—are the same properties that make it erode and that make it sensitive to dilution in a well-perfused site; injectability and long residence are, therefore, in tension, and every platform in Table 6 represents a particular resolution of that tension rather than an escape from it. Second, mesh size is a weaker design lever than the literature implies. It controls release effectively only over the narrow range where the solute radius approaches the mesh dimension; below that range, the solute diffuses essentially freely, and above it, the solute is excluded, and release becomes erosion-limited [12,86]. For small-molecule cargo in a conventional hydrogel, the system is almost always in the first regime, which is why bulk hydrogels alone rarely achieve sustained small-molecule release and why the nanoparticle compartment of Section 5 is added.

## 5. Hybrid Nanoparticle–Hydrogel Composite Systems

The defining drug-delivery story of the past five years is not that nanoparticles displaced hydrogels, or vice versa, but that they have been combined. Embedding nanoparticles within hydrogel matrices—or, conversely, using nanoparticles as crosslinkers of polymer networks—produces hybrid nanocomposite hydrogels that retain the advantages of each component while compensating for the principal weaknesses of either one [11,12,15,16,17,103]. This section examines the design rationale (Section 5.1), the principal hybrid architectures (Section 5.2), and the influence of network architecture on cargo retention and release (Section 5.3).

### 5.1. Design Rationale: Why Hybridize?

A pure hydrogel solves three problems poorly: it loads hydrophobic APIs inefficiently because of its hydrophilic interior; it suffers from initial burst release because mesh-confined drug equilibrates with the surrounding fluid before the polymer network can degrade; and it provides only limited possibilities for stimuli-responsive triggering with single-component networks [11,12,15]. A pure nanoparticle, conversely, solves a different set of problems poorly: it accumulates briefly at its target before being cleared; it cannot deliver months-long sustained exposure from a single dose; and, when injected systemically, it must navigate biological barriers that frequently divert >95% of the dose to the mononuclear phagocyte system [4,7,9,11].

Combining the two yields a system with at least five complementary advantages [11,12,15,16,17]: (i) hydrophobic drugs are pre-encapsulated in the NP compartment and protected from the hydrophilic gel; (ii) the gel suppresses NP burst release and confines NPs at the implantation site for weeks to months; (iii) two or more cargoes—a small molecule in NPs, a biologic in the matrix, or two NPs with different release rates—can be delivered with programmable, multi-phasic kinetics; (iv) the NPs serve as anchor points that improve hydrogel mechanics (storage modulus and toughness) and confer stimuli responsiveness (photothermal heating from gold or iron-oxide NPs, as well as pH-triggered MSN gating); and (v) upon slow gel erosion, drug-loaded NPs are released into the local tissue, where they retain their targeting and intracellular delivery capabilities. Recent reviews note that this complementarity has driven a 4–5-fold increase in nanocomposite hydrogel publications between 2020 and 2025 [11,12,15].

### 5.2. Principal Hybrid Architectures

**Figure 5:**
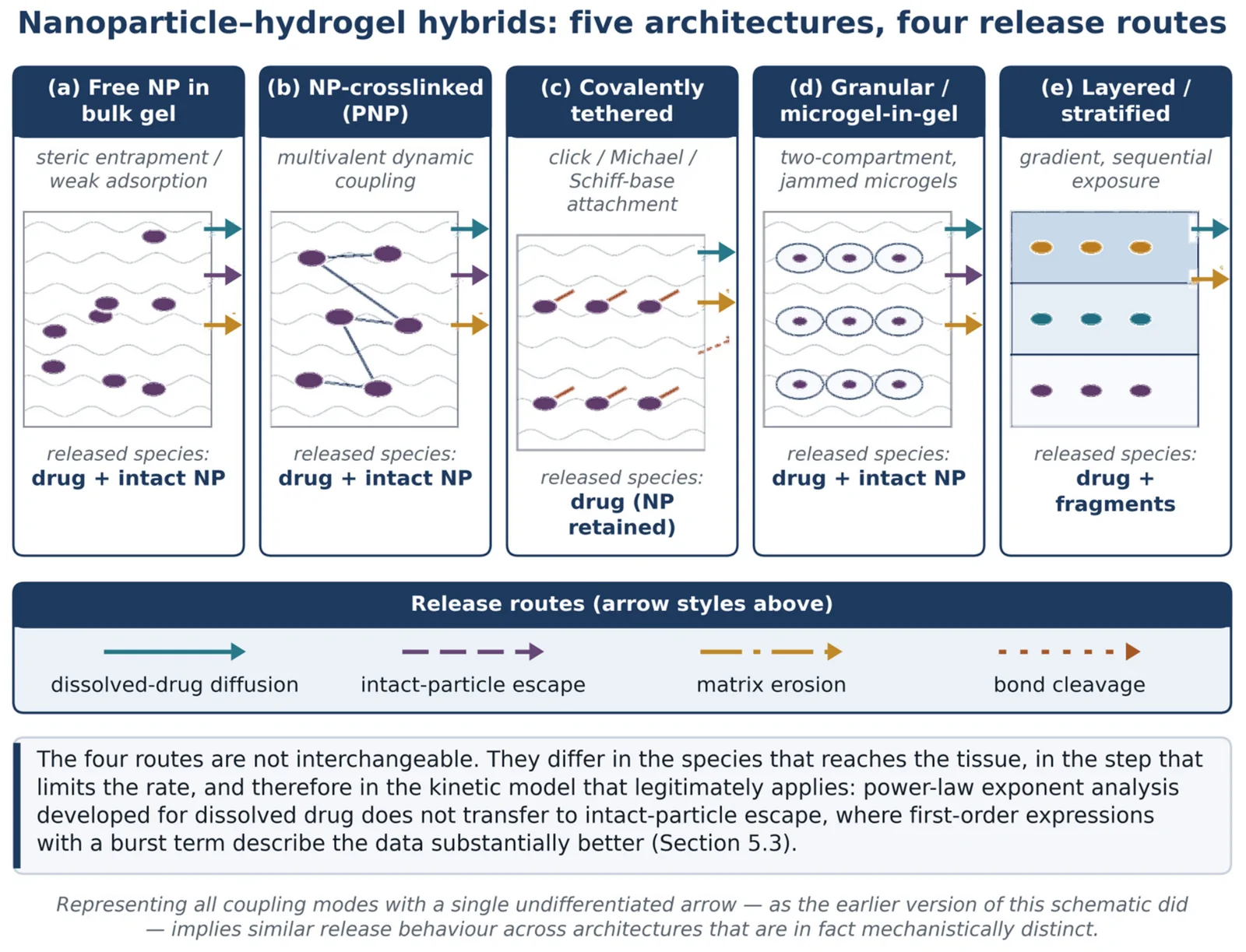
Nanoparticle–hydrogel hybrids: five architectures and the four release routes available to them. (**a**) Free NP in bulk gel; (**b**) nanoparticle-crosslinked (PNP) hydrogel; (**c**) covalently tethered NP hybrid; (**d**) granular or microgel-in-gel composite; and (**e**) layered or stratified construct. Arrow styles distinguish dissolved-drug diffusion, intact-particle escape, matrix erosion and bond cleavage; the released species is stated for each architecture. Representing all coupling modes with a single undifferentiated arrow implies similar release behavior across architectures that are mechanistically distinct. Original figure created by the authors using Matplotlib 3.10.8 (Python 3.12.3).

Hybrid systems can be classified by how the nanoparticles are integrated into the polymer network (Figure 5; Table 7) [11,15,16,17,103].

*Free-NP hydrogels* are the simplest case: pre-formed NPs (liposomes, PLGA particles, MSNs, and SPIONs) are physically dispersed in a polymerizing or self-assembling hydrogel precursor. The NPs occupy the gel interstices, are retained sterically as long as their diameter exceeds the network mesh size, and are released as the gel degrades or swells [16,18]. Drug egress proceeds along two parallel routes: diffusion of free drug through the gel mesh, and slower release of intact NPs that subsequently liberate their payload in the surrounding tissue [16,17].

*Nanoparticle-crosslinked hydrogels (PNP gels)* exploit specific or non-specific interactions between NP surfaces and polymer chains to form the crosslinks themselves. The paradigmatic example is the polymer–nanoparticle network of Appel and colleagues, in which dodecyl-modified hydroxypropyl methylcellulose adsorbs onto PEG-PLA NPs through multivalent hydrophobic association [11,33,98]. Other variants include host–guest PNP networks (cyclodextrin-decorated NPs with adamantane-modified polymers), boronate ester PNP networks (diol-bearing NPs with phenylboronic acid polymers, which are intrinsically glucose-responsive) and electrostatically assembled networks [11,15]. Because the crosslinks are dynamic, these gels are shear-thinning and self-healing—injectable through standard 25–30G needles—and their erosion is governed by the unbinding kinetics of the NP–polymer association.

*Covalently tethered NPs* are anchored to the polymer backbone via click chemistry, Michael addition or Schiff-base bonds, so that NPs remain in the matrix until the gel itself degrades [11,15]. This architecture maximizes NP retention and is favored for situations requiring spatial confinement of imaging contrast agents or when premature NP release would compromise targeting.

*Microgel-in-gel and granular composites* are constructed by suspending pre-formed microgels or nanogels in a secondary continuous network or by jamming microgels together to form a granular hydrogel with interconnected porosity. The result is a hierarchical material in which the cargo can be partitioned between micro- and macro-compartments and released at distinct rates [11,17,30,95].

*Layered or stratified hybrids* (printed multilayer films, core–shell injectable depots, and microneedle patches with NP gradients) extend these concepts into spatially programmed delivery, in which different drugs or NPs are loaded at different depths of the construct and become accessible sequentially [12,17].

Classification by released species: The five architectures above are conventionally distinguished by how the nanoparticle is coupled to the network. For release behavior, a second and more informative distinction is available: what actually leaves the construct, and which transport step limits it (Figure 5; Table 7). Four released species are possible. Dissolved drug that has escaped the nanoparticle compartment diffuses through the network mesh, limited by mesh size relative to solute radius. Drug may be liberated from a nanoparticle that remains within the matrix, in which case, the rate is set by intra-particle release and only secondarily by the gel. An intact, still-loaded nanoparticle may escape the network, limited by interfacial unbinding and by matrix erosion rather than by mesh geometry, and will liberate its cargo in the surrounding tissue with its targeting and intracellular delivery capabilities preserved. Finally, fragments of a degrading matrix may carry adsorbed cargo away with them.

The distinction is not taxonomic. These four routes differ in their kinetic signature, in the assay required to detect them, and in the biological consequence of the release event, and a construct that appears to show simple sustained release may exhibit any of them or several at once. Covalently tethered architectures are the only ones that exclude intact particle escape by design, which is why they are chosen where premature particle release would compromise targeting or imaging accuracy [11,15]. Conversely, free-NP and polymer–nanoparticle constructs release both drugs and particles in parallel, so that a measurement of total drug in the receiving medium conflates two processes with different rate laws [16,17]. Section 5.3 examines what the available data show about the particulate route specifically, which is the least studied of the four.

### 5.3. Influence of Network Architecture on Retention and Release

Across all architectures, cargo retention and release kinetics are governed principally by the NP–polymer interfacial chemistry—the covalent, coordination, hydrogen bonding or electrostatic coupling between particle and network—together with the NP loading and the degradation rate of the matrix; the NP-to-network size ratio sets an outer bound on what can escape, but is not reliably the dominant lever [11,12,16,17,103].

A 2025 study by Glomm and colleagues addressed a question the nanocomposite literature has largely left open: how the nanoparticles themselves—rather than a dissolved cargo—escape the gel [16]. Dye-loaded polystyrene nanoparticles (200 nm) were incorporated at up to 10% *w*/*w* into internally gelled alginate and alginate–poly(ethylene oxide) networks and followed for approximately 1200 h at 37 °C. Cumulative release remained low throughout (of the order of 10%) and was inversely proportional to nanoparticle loading; partial least-squares regression identified time and nanoparticle concentration as the dominant predictors of release, with alginate concentration being less influential by more than an order of magnitude, although reducing alginate from 1.5 to 0.75 wt% did modestly improve release efficiency by enlarging the mesh [16]. Of six kinetic models tested, a modified first-order expression incorporating a burst term fit every system best (R^2^ ≈ 0.98–0.99), whereas the Korsmeyer–Peppas power law fit worst (R^2^ ≈ 0.72–0.90), a useful caution against transplanting the exponent analysis of Section 2.3 from molecular to particulate release [16,29]. Mechanistically, the barrier appears interfacial rather than purely architectural: nanoparticle-loaded gels resisted EDTA dissolution entirely, indicating that the particles associate with the alginate chains and act as additional crosslinkers, so that raising the particle load densifies the very network that must release them [16]. A complementary 2025 review by Stevanović et al. reached a convergent conclusion from the materials side: nanoparticles act as multifunctional crosslinking nodes that raise network density and improve load transfer between the inorganic and polymeric phases, and it is this interface—assembled from covalent, coordination, hydrogen-bonding or electrostatic interactions—that simultaneously governs mechanical reinforcement, swelling behavior and release, with well-designed interfaces suppressing burst effects [12]. The reported gains are strongly system-dependent rather than generic: gold nanoparticles roughly double the tensile strength of PNIPAM networks while conferring ≈85% self-healing within two minutes under near-infrared irradiation; TiO_2_–carbon-nanotube fillers raise the compressive modulus and puncture resistance of polyacrylamide; and mesoporous silica nanoparticles in GelMA/PEGDA suppress burst release and sustain IGF-1 delivery beyond 144 h [12]. Charge interactions are equally important: positively charged NPs retain longer in anionic hyaluronate or alginate networks than in neutral PEG networks, while ROS-cleavable thioketal linkers between NP and polymer convert the entire assembly into an oxidative-stress-responsive system [11,15].

For sustained-release applications spanning weeks to months, no comparable design rule has yet emerged for the hybrid case. Reviewing the field in 2025, Fan and colleagues conclude that the interactions between embedded nanoparticles and the surrounding network remain poorly characterized and understood—in part, because studies overwhelmingly track the release of the loaded drug rather than the behavior of the particle itself—and identify this as an open gap rather than a settled design problem [11]. What the literature currently supports is directional rather than numerical: retention improves as NP–polymer coupling shifts from steric entrapment towards hydrogen-bonding, electrostatic or covalent association [11,12,15]; denser, more highly crosslinked networks with a smaller mesh slow the escape of both drug and particle [11,17]; and release can be tuned through polymer degradation rate, crosslinking density and NP surface chemistry, although predictable kinetics still require empirical fitting system by system [15]. Prescriptive figures—a defined mesh-size-to-particle-diameter ratio, or a matrix degradation half-life matched to a target release duration—are not established by the available comparative data and are not asserted here (cf. Section “Limitations of This Review”). The best-characterized long-acting example in this class is a supramolecular PNP hydrogel delivering bimatoprost by intravitreal injection in rabbits, in which the depot degraded slowly and maintained detectable vitreal drug levels for up to eight weeks following a single administration, alongside a mild foreign-body response [33].

### 5.4. Influence of Matrix Chemistry on Release from Embedded Nanoparticles

The preceding section considered how the network governs the escape of the particle. The complementary question—how the choice of matrix chemistry modifies release from the nanoparticles themselves, while they remain embedded—is distinct, less well studied, and practically important, because in most hybrid constructs, the majority of the cargo is liberated from particles that never leave the gel.

Three mechanisms are documented. The first is chemical: the matrix sets the local microenvironment that the embedded particle experiences, and this can either enable or suppress the particle’s own trigger. A pH-responsive particle embedded in a polyanionic network such as alginate or hyaluronate sits in a locally more acidic Donnan environment than the bulk medium, so its apparent trigger threshold shifts; conversely, the acidic microclimate generated inside a bulk-eroding PLGA particle can be buffered by a surrounding network, altering the autocatalytic hydrolysis that drives its characteristic triphasic profile [45,46]. Where the matrix and the particle respond to the same cue, the two responses are in series rather than independent, and the slower of the two governs. The second is electrostatic and interfacial: positively charged particles are retained substantially longer in anionic hyaluronate or alginate networks than in neutral PEG networks, and the same attraction that retains them also restricts the local mobility of released cargo near the particle surface [11,15]. Interfacial coupling assembled from covalent, coordination, hydrogen-bonding or electrostatic interactions simultaneously governs mechanical reinforcement, swelling behavior and release, with well-designed interfaces suppressing burst effects [12].

The third is physical and is the one most often overlooked: nanoparticles do not sit passively in the network but act as additional crosslinking nodes, so that raising the particle load densifies the very matrix through which cargo must travel. This is why cumulative release can fall as nanoparticle loading rises, and why particle-loaded alginate gels resist chelation that dissolves the unloaded network entirely [12,16]. The reported effects are strongly system-dependent rather than generic: gold nanoparticles roughly double the tensile strength of PNIPAM networks while conferring approximately 85% self-healing within two minutes under near-infrared irradiation; TiO_2_–carbon-nanotube fillers raise the compressive modulus and puncture resistance of polyacrylamide; and mesoporous silica nanoparticles in GelMA/PEGDA suppress burst release and sustain IGF-1 delivery beyond 144 h [12]. No general design rule relating matrix chemistry to release from embedded particles has yet emerged, and the reason is methodological as much as material: studies overwhelmingly track the drug rather than the particle, so the two contributions are rarely separated [11]. Reporting them separately, as Section 2.3 argues for release testing generally, is the precondition for such a rule.

### 5.5. Comparative Assessment of Hybrid Architectures

Comparative assessment of hybrid architectures. Reading Table 7 by released species rather than by coupling chemistry makes the design trade-off explicit. Retention and controllability move together, and both move against responsiveness. Covalent tethering maximizes retention and eliminates the particulate release route, but it also fixes the particle in a matrix whose degradation then becomes the sole release lever. Polymer–nanoparticle networks sit at the opposite extreme: their dynamic junctions make them injectable and their erosion tunable, but the same junctions release intact particles whose subsequent fate is not controlled by the formulation. Free-NP dispersions are the simplest to manufacture and the least predictable because retention depends on a steric relationship between particle diameter and mesh that is itself altered by the particles. Granular assemblies uniquely decouple the two compartments, permitting genuinely independent release rates from the microgel and interstitium, at the cost of mechanical fragility. Layered constructs are the only architecture that programs release in space rather than only in time and are, correspondingly, the most difficult to manufacture reproducibly [11,12,15,17].

## 6. Stimuli-Responsive Release Mechanisms in Detail

**Figure 6:**
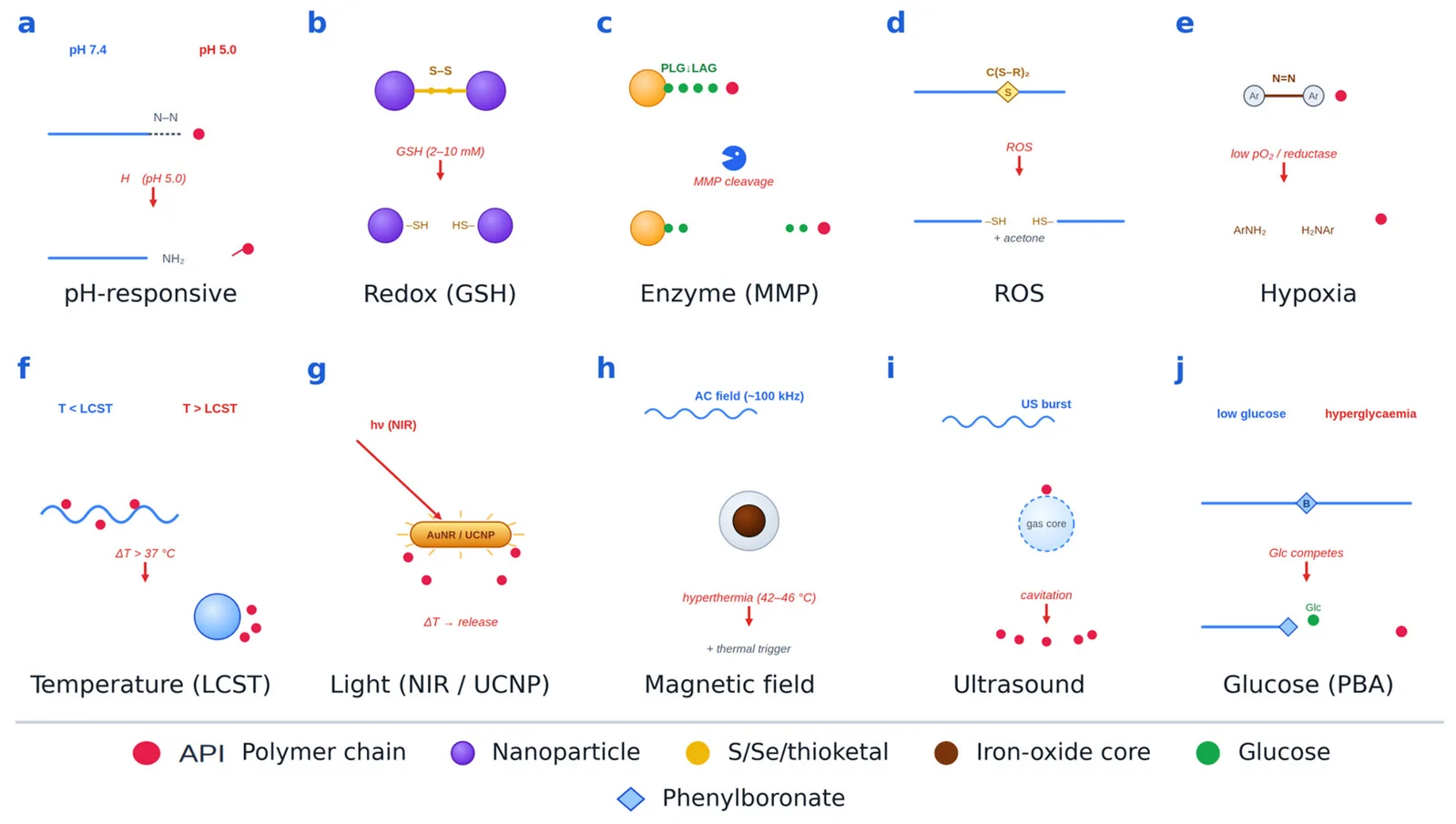
Principal stimuli-responsive chemistries and their release-triggering mechanisms. (**a**) pH: protonation of charge-reversal amines and acid cleavage of hydrazone/acetal linkages; (**b**) redox: GSH-driven cleavage of disulfide and diselenide bonds; (**c**) enzymes: proteolytic cleavage of MMP-substrate peptide spacers; (**d**) ROS: thioketal fragmentation by elevated reactive oxygen species; (**e**) hypoxia: bioreduction of azobenzene and nitroaromatic groups; (**f**) temperature: LCST-driven coil-to-globule transition above 37 °C; (**g**) light: NIR-induced photothermal heating of plasmonic/upconversion nanoparticles; (**h**) magnetic field: alternating-field hyperthermia of SPIONs; (**i**) ultrasound: microbubble cavitation and acoustic droplet vaporization; and (**j**) glucose: competitive PBA–glucose binding displacing diol crosslinks under hyperglycemia. Original figure created by the authors using Matplotlib 3.10.8 (Python 3.12.3).

The conceptual classification of stimuli (Section 2.4) becomes useful only when paired with the molecular chemistry that turns each stimulus into a release event. This section dissects the ten mechanism families that dominate the contemporary literature—pH, redox, enzyme, ROS, hypoxia, temperature, light, magnetic field, ultrasound and glucose—and closes with multi-stimuli systems. For each class, we identify the responsive moiety, the physical or chemical event it triggers, the achievable on-state contrast against the off-state baseline, and the principal therapeutic context (Figure 6; Table 8).

### 6.1. pH-Responsive Systems

The protonation state of carrier components is the most exploited endogenous switch. Two main strategies dominate. Acid-cleavable bonds—hydrazones, β-thiopropionates, acetals, ketals, *cis*-aconityl and orthoester linkages—hydrolyze selectively at lysosomal pH (4.5–5.5) but remain stable at extracellular pH 7.4, allowing drug–polymer conjugates to release cargo only after cellular uptake [5,6,34,47,104]. Hydrazone linkages between doxorubicin and polymeric backbones, pioneered by Ulbrich and now in several clinical-stage formulations, increase intracellular release rate > 20-fold relative to neutral pH [5,104]. Charge-reversal polymers—which bear amino groups (PDPA and PDEAEMA), imidazoles or β-amino esters—protonate as pH falls below their pKa (typically 5.5–6.8), inducing micelle disassembly, polymer swelling, or “proton-sponge”-mediated endosomal escape [5,47,120]. A 2025 *Int J Pharm X* study by Yu et al. demonstrated ultra-sensitive pH-responsive injectable hydrogels that achieve a near-step-function release transition between pH 7.4 and 6.5, suitable for deep-tumor delivery [89].

### 6.2. Redox-Responsive Systems

The 100–1000-fold gradient between intracellular GSH (2–10 mM) and plasma GSH (~2 µM) is the molecular foundation for redox-responsive carriers [105,106]. Disulfide bonds (S–S)—incorporated into polymer backbones, side chains, and drug–polymer linkers, or as crosslinkers—are stable in circulation and rapidly cleaved by thiol-disulfide exchange in the cytosol, releasing cargo in seconds to minutes after entering the cell [105,106,107]. Diselenide bonds (Se–Se), with a lower bond dissociation energy than S–S, are even more sensitive to GSH and additionally responsive to elevated ROS, making them dual-redox triggers [105,106,108]. Trisulfide (S–S–S) bonds, introduced into nanocarriers in 2024, exhibit ~five-fold faster cleavage than disulfides at equivalent GSH levels [106]. A 2024 *J Nanobiotechnol* systematic review of redox-manipulating carriers cautioned that many published systems achieve incomplete release at physiologically realistic intracellular GSH concentrations (1–10 mM rather than the 50–100 mM often used in vitro), and identified crosslinking density, polymer hydrophobicity and NP size distribution as critical but frequently under-reported variables [106]. Recent platforms exemplify the field: a 2024 chitosan–heparin micellar system bearing lipoic-acid-linked S–S bridges reduced doxorubicin release at pH 7.4 to <20% at 6 h while increasing it >three-fold at pH 5.0 + 10 mM GSH [105]. Cisplatin-loaded diselenide-bridged mesoporous organosilica nanoparticles reached 60% in tumors and <10% in plasma within 24 h, simultaneously depleting tumor GSH and amplifying oxidative stress [108].

### 6.3. Enzyme-Responsive Systems

Enzymes overexpressed in disease tissue convert biochemical recognition into physical cargo release [5,47,112]. Matrix metalloproteinases (MMP-2, MMP-9, and MMP-7), elevated 5–100-fold in tumor stroma and infarcted myocardium, cleave defined peptide sequences (PLGLAG, GPLGVRG, and GPQG↓IWGQ) inserted between hydrophilic and hydrophobic blocks of self-assembled nanocarriers; cleavage destabilizes the carrier and releases payload [112,113]. The Christman laboratory’s MMP-responsive peptide–polymer amphiphile micelles, which aggregate at the infarcted myocardium following IV administration and are retained for up to 28 days, exemplify the disease-localizing potential of this strategy [113]. Hyaluronidase (HAase), overexpressed in many solid tumors, degrades hyaluronic acid (HA) coatings or HA-based hydrogels, releasing entrapped NPs and drug [5,47]. Esterases, cathepsin B and L, phosphatases and prostate-specific antigen further extend the enzymatic toolkit, with cathepsin-cleavable Val-Cit dipeptide linkers now standard in approved antibody–drug conjugates and increasingly used in nanomedicines [5,47,112]. Bacterial enzymes (lipases and gelatinases) provide infection-selective release in antimicrobial dressings [11,15].

### 6.4. ROS- and Hypoxia-Responsive Systems (And the Oxidative–Reductive Paradox)

Reactive oxygen species (H_2_O_2_, singlet oxygen, superoxide, and hydroxyl radical) accumulate in tumor, inflamed and ischemic tissues at concentrations of 50–100 µM (vs. ~20 nM in healthy tissues) and are exploited by thioketal, arylboronate, selenium and ferrocene linkages that fragment upon oxidation [47,109,110,111]. Thioketal (TK) bonds—degraded by ^1^O_2_, OH• and ONOO^−^ into acetone and thiols—are the most popular ROS trigger, with paclitaxel- and camptothecin-TK prodrug nanoparticles now demonstrating chemo-photodynamic synergy in colon cancer models [109,110]. Arylboronates oxidize to phenols and have been used to mask amines and hydroxyls for ROS-gated activation [47,111]. Hypoxia-responsive systems exploit reductase enzymes (azoreductase, nitroreductase, and NADPH:cytochrome P450 reductase) overexpressed under low pO_2_. Azobenzene linkages undergo bioreduction to amines under hypoxia, cleaving polymer–drug conjugates; nitroaromatic moieties (in tirapazamine-style prodrugs) and 2-nitroimidazoles are likewise reduced selectively in hypoxic cells [47,114]. Crucially, photodynamic therapy itself depletes oxygen and amplifies hypoxia, enabling self-reinforcing PDT–hypoxia-activated combinations [114].

It is worth flagging an under-discussed mechanistic tension: although ROS and hypoxia are both elevated in tumors, they are mechanistically opposite stimuli—oxidative versus reductive—yet they often co-exist in the same poorly perfused tumor core. A carrier engineered to fragment under high ROS will be in chemical conflict with one engineered to be reduced under low pO_2_, and a single carrier carrying both motifs (e.g., a thioketal–azobenzene hybrid) faces an effectively non-orthogonal trigger landscape unless the cleavage chemistries are kinetically separated. Practically, this paradox argues for sequential rather than truly simultaneous dual-trigger logic and is one reason that AND-gated systems combining an endogenous (e.g., pH or enzyme) and an exogenous (e.g., light or ultrasound) cue have advanced more rapidly than purely endogenous AND combinations [25,26,34,106,109].

### 6.5. Thermo-Responsive Systems

Polymers with a lower critical solution temperature (LCST)—most prominently poly(*N*-isopropylacrylamide) (PNIPAM; LCST ≈ 32 °C), poloxamer 407 (exhibiting sol–gel transition near 25 °C in a concentrated solution), elastin-like polypeptides, and poly(*N*-vinylcaprolactam)—undergo entropy-driven dehydration and chain collapse above their LCST, expelling water and entrapped drug [12,89,115]. Tuning LCST close to body temperature (37 °C) by copolymerization with hydrophilic comonomers enables in situ gelation at the injection site (poloxamer 407 sols injected at 4 °C; gel injected at 37 °C) and on-demand release when local hyperthermia (40–43 °C, achieved by external sources or by photothermal/magnetic NPs) drives a sharp volume-phase transition [115]. PNIPAM-based microgels in particular show release accelerations of 3–10× upon mild hyperthermia [115].

### 6.6. Photo-Responsive Systems

Light triggers release via three orthogonal mechanisms [22,116]. Photoisomerization of *azobenzene* (UV/visible) and *spiropyran/merocyanine* changes molecular geometry and polarity, opening hydrogel mesh size or expelling drug from cyclodextrin host–guest complexes. Photocleavage of *o*-nitrobenzyl-, coumarinyl- and *p*-methoxyphenacyl-protecting groups breaks polymer–drug bonds with single-photon UV or two-photon NIR excitation [22,116]. Photothermal conversion by gold nanorods/cages, copper sulfide NPs, MoS_2_ nanosheets, polydopamine or porphyrin assemblies generates local heat (≥45 °C) under NIR (700–900 nm or 1000–1100 nm (second-window)) light, which couples to thermo-responsive networks for combined release and hyperthermia [6,22]. Upconversion nanoparticles (UCNPs) convert tissue-penetrating NIR (980 nm) into UV/visible light, enabling photocleavage chemistry at depth without UV phototoxicity [6,22]. The principal limitation—a penetration depth of <1 cm for visible light and 2–3 cm for NIR light—restricts photo-responsive systems to skin, eye, oral cavity, intra-operative use, and superficial tumors.

### 6.7. Magnetic- and Ultrasound-Responsive Systems

Iron oxide/SPION-based systems respond to two distinct magnetic cues. Static magnetic fields direct NP accumulation to a target site; alternating magnetic fields (100 kHz–1 MHz; 5–25 kA m^−1^) generate magnetic-hyperthermia heating (42–46 °C) that triggers release from thermo-responsive shells while simultaneously providing tumor ablation [23,66,117]. NanoTherm^®^ (MagForce AG, Berlin, Germany) was the first magnetic-hyperthermia product to obtain a CE mark, granted in 2010 as a medical device rather than as a medicinal product, for recurrent glioblastoma. Its subsequent history is instructive: clinical uptake remained confined to a small number of EU centers, the sponsor entered insolvency proceedings in 2022, and the current developer states that the prior CE marking has been discontinued and that the system is an investigational device not approved for commercial clinical use in any jurisdiction. The technology, therefore, holds no active marketing authorization at the time of writing, and it is represented accordingly in Table 10 and Figure 8 [23,117,121]. Ultrasound (US)-responsive systems exploit thermal, mechanical (cavitation and shear) and acoustic radiation effects [42,118]. Microbubbles and phase-change perfluorocarbon nanodroplets undergo acoustic vaporization; piezoelectric polymers convert mechanical oscillation into local electric fields; and US-induced sonoporation transiently permeabilizes cell membranes for enhanced uptake [118]. Focused-ultrasound–MRI integration permits sub-millimeter-precise targeting of the acoustic field in deep-seated tissue, and the same focusing capability is used to open the blood–brain barrier transiently and locally, so that a co-administered agent can enter the intracranial compartment [42]. The latter is barrier-opening, or facilitated delivery, and not carrier-triggered cargo release. None of the work cited here demonstrates that the acoustic field simultaneously liberates cargo from a carrier at the insonated site, and the capability is accordingly classified as externally assisted delivery throughout this review (Section 2.1, Table 2).

### 6.8. Glucose-Responsive Systems

Glucose-responsive insulin delivery is the central goal of an “artificial pancreas” pharmaceutical [21,31,32]. Three molecular strategies dominate. Glucose oxidase (GOx) converts glucose to gluconic acid + H_2_O_2_, lowering local pH and generating ROS; embedding GOx in a pH- or ROS-responsive matrix translates hyperglycemia into insulin release [31,32]. Phenylboronic acid (PBA) forms reversible boronate esters with diols, including glucose; in hyperglycemic conditions, glucose competes with diol crosslinkers in a hydrogel, weakening the network and accelerating insulin release [21,31,32]. Concanavalin A (ConA), a lectin that binds to glucose with high affinity, is used as a sugar-recognizing crosslinker in glucose-responsive gels, although immunogenicity has tempered its translation [21]. A 2024 *Theranostics* paper reported an orally delivered, glucose-responsive insulin nanoparticle that used a glucose oxidase/phenylboronic ester system to release bioactive insulin in response to the acidity and hydrogen peroxide generated from glucose oxidation, achieving glucose control comparable to subcutaneous insulin in a type 1 diabetes model [119].

### 6.9. Multi-Stimuli Systems and the Criteria for AND-Gated Behavior

**Figure 7:**
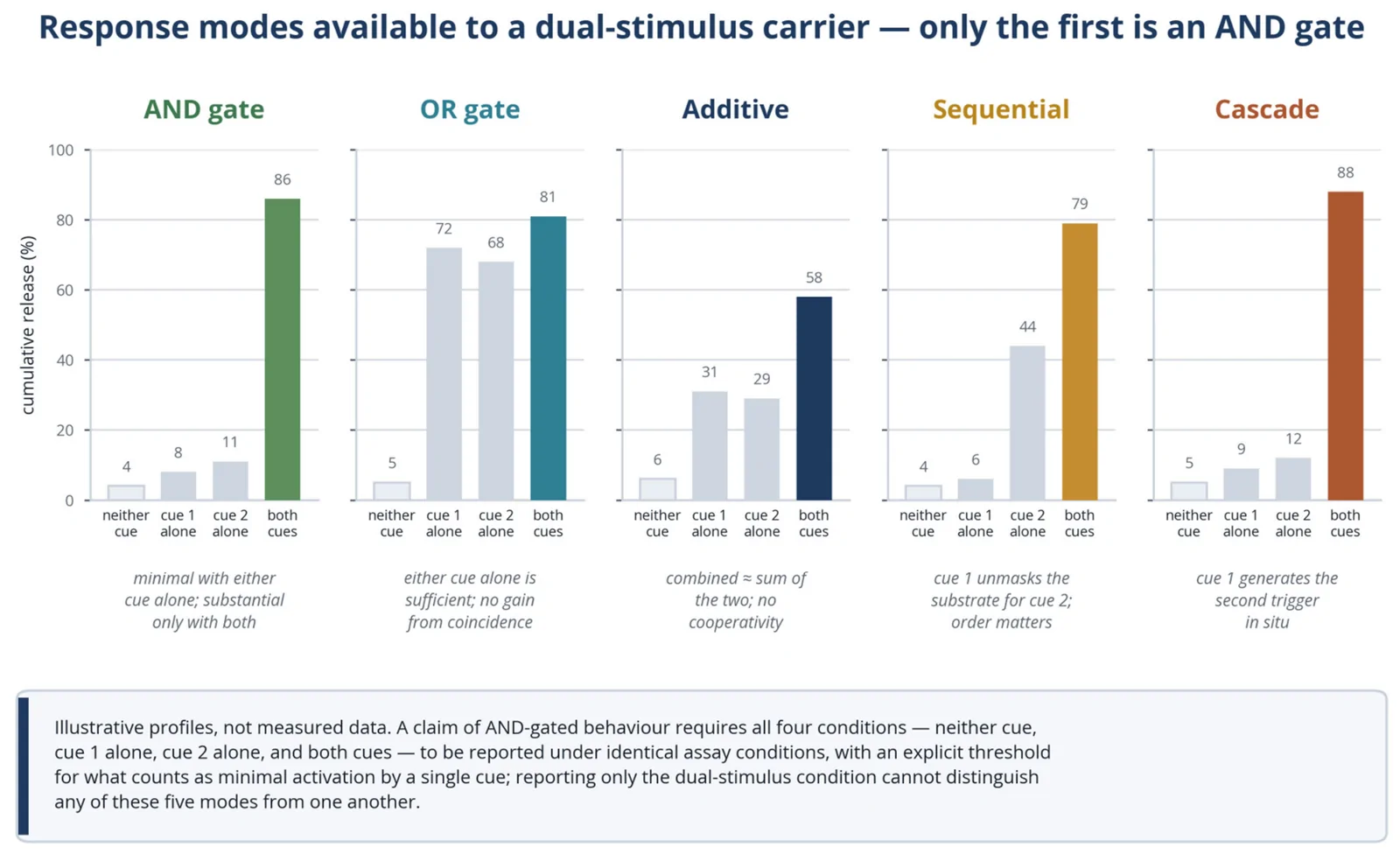
Response modes available to a dual-stimulus carrier. Only the first is an AND gate. Profiles are illustrative rather than measured. Establishing AND-gated behavior requires release to be reported under all four conditions—neither cue, cue 1 alone, cue 2 alone, and both cues—under identical assay conditions, with a pre-stated threshold for minimal activation; dual-stimulus data alone cannot distinguish these five modes. Original figure created by the authors using Matplotlib 3.10.8 (Python 3.12.3).

A single endogenous trigger rarely discriminates perfectly between diseased and healthy tissue, and combining two or more cues is, therefore, an established strategy for sharpening spatial selectivity [25,26,34]. The term multi-stimuli, however, describes only the number of cues a carrier responds to; it says nothing about the logic relating them, and it is not a synonym for AND-gated behavior. A dual-responsive system may exhibit any of at least five distinct response modes (Figure 7), which have different selectivity consequences and require different experimental evidence to establish.

In an AND gate, either cue alone produces minimal release, and substantial release occurs only when both are present; selectivity is genuinely multiplied, and off-target activation requires coincidence. In an OR gate, either cue alone is sufficient; the system is more likely to fire, not less, and selectivity is reduced relative to either single-stimulus parent. In an additive system, the response to both cues approximates the sum of the individual responses with no cooperativity, so single-stimulus activation remains substantial. In a sequential system, the order matters: the first cue unmasks a substrate or exposes a linkage that the second then acts on, so the pair is effective only in one order. In a cascade, the first cue generates the second trigger in situ—as when glucose oxidase converts glucose into gluconic acid and hydrogen peroxide, and a pH- or ROS-responsive matrix responds to those products rather than to glucose itself (Section 6.8)—so what appears to be dual-responsiveness is one trigger acting through an intermediate.

Establishing AND-gated behavior, therefore, requires a specific experimental design: release must be measured under all four conditions (neither cue, cue 1 alone, cue 2 alone, or both cues), under identical assay conditions and with an explicit, pre-stated threshold defining what counts as minimal activation by a single cue. Reporting only the dual-stimulus condition, which is common, cannot distinguish an AND gate from an OR gate, an additive system or a cascade, and a claim of AND-gating unsupported by the single-stimulus controls should be read as a claim of dual-responsiveness only. Because the magnitude of every one of these conditions depends on the release assay used (Section 2.3), single-stimulus controls run under different conditions from the dual-stimulus experiment do not satisfy this requirement.

Judged against this criterion, dual thermo- and pH-responsive polymer nanoparticle assemblies reported by Pytlíková and colleagues do behave as an AND gate: release was negligible under either trigger alone and exceeded 85% within 4 h when both were applied [25]. Frequently reported combinations include pH with redox (charge-reversal coatings around disulfide-crosslinked cores), pH with temperature (PNIPAM-co-acrylic-acid copolymers), pH with light (near-infrared photothermal triggering of pH-responsive disassembly), redox with ROS (diselenide–thioketal hybrid linkers) and enzymes with light [25,26,34,106,109], but the logic of most of these has not been characterized to the standard above. It is also worth noting that combinations pairing an endogenous cue with an exogenous cue are structurally better suited to AND gating than purely endogenous pairs because the exogenous cue is under operator control and can be withheld, which makes the single-stimulus control trivial to perform and the off-state genuinely off. Purely endogenous pairs face the additional difficulty noted in Section 6.4, that co-elevated cues in the same lesion may be chemically antagonistic. A 2025 review documented that more than half of newly reported anticancer carriers in 2023–2024 used dual or triple stimuli [34]; how many of them are AND-gated in the sense defined here has not been established by the available reporting.

A counter-example makes the distinction concrete, and it is instructive precisely because its authors reported the controls that allow the classification to be made at all. Wei and colleagues synthesized salecan/poly(N,N-diethylacrylamide-co-methacrylic acid) semi-interpenetrating networks, in which the poly(N,N-diethylacrylamide) component confers thermo-responsiveness and the methacrylic acid units confer pH responsiveness through protonation of carboxylate below their pK_a_ of 4.9, and characterized doxorubicin release under all four combinations of temperature (25 and 37 °C) and pH (7.4 and 4.0) [122]. Each cue was individually effective: raising the temperature alone markedly increased cumulative release, and lowering the pH alone did likewise. Applying both together, however, produced release only marginally above that obtained with the more effective single cue, and below the sum of the two individual increments. On the criteria set out above, this is not an AND gate but a sub-additive, effectively OR-type response: a system in which either trigger is sufficient, so that selectivity is lower than that of either single-stimulus parent rather than higher.

The chemistry supplies the explanation, and with it, two design rules. Both cues act on the same physical event—collapse of the network above its volume phase transition and expulsion of the entrained aqueous phase, which carries dissolved drug with it—so once the first cue has driven that collapse, the second has little left to act upon. Genuine AND gating, therefore, requires the two cues to operate on separable elements of the construct, one governing linker or gate integrity and the other governing matrix permeability, rather than converging on a single shared transition. Second, the transition must itself be sharp. Poly(N,N-diethylacrylamide) undergoes a continuous phase transition spread over an interval of roughly 15 °C, in contrast with the sharp transition of poly(N-isopropylacrylamide) near 32 °C [122], so partial activation at intermediate temperatures is intrinsic to the material, and a well-defined off-state is not available to it. This example also illustrates the evidential standard argued for in Section 2.3: the release claims are supported by independent swelling and deswelling kinetics, scanning electron micrographs of pore structure and rheological measurement of the storage modulus, so the mechanism is established by convergent measurement rather than by curve fitting alone [122].

Stimulus-specific limitations: The translational obstacles above are common to all complex carriers. A second set is specific to the trigger chosen, and because these are rarely compared side by side, they are set out in Table 9. Three groupings emerge. Endogenous chemical triggers (pH, redox, ROS, enzyme, and hypoxia) suffer principally from biological variability: the cue is present in healthy tissue at lower but non-zero levels, its magnitude varies between patients and between regions of the same lesion, and the concentrations used to demonstrate responsiveness in vitro frequently exceed those achievable in vivo, a discrepancy documented explicitly for glutathione, where many published systems are characterized at 50–100 mM against a physiological intracellular range of 1–10 mM [106]. Their toxicological liabilities arise from the cleavage products themselves: thioketal fragmentation liberates acetone and thiols, and selenium-containing linkers raise questions of chronic exposure that are not resolved by acute studies [109,110]. Exogenous physical triggers invert the profile: activation is under operator control, and the off-state is genuinely off, but penetration depth constrains applicability—under 1 cm for visible light and 2–3 cm for near-infrared—and the safety concerns are thermal and mechanical rather than chemical, encompassing thermal injury to adjacent tissue during photothermal or magnetic hyperthermia and inertial cavitation during ultrasound exposure [6,22,23,42,118]. Inorganic carriers add a third and separate liability: the long-term fate of the non-degradable component and the possibility of metal-ion release [19,20,66,69], while PEGylated constructs of any trigger class face the immunogenicity problem set out above [123,124].

## 7. Therapeutic Applications

**Figure 8:**
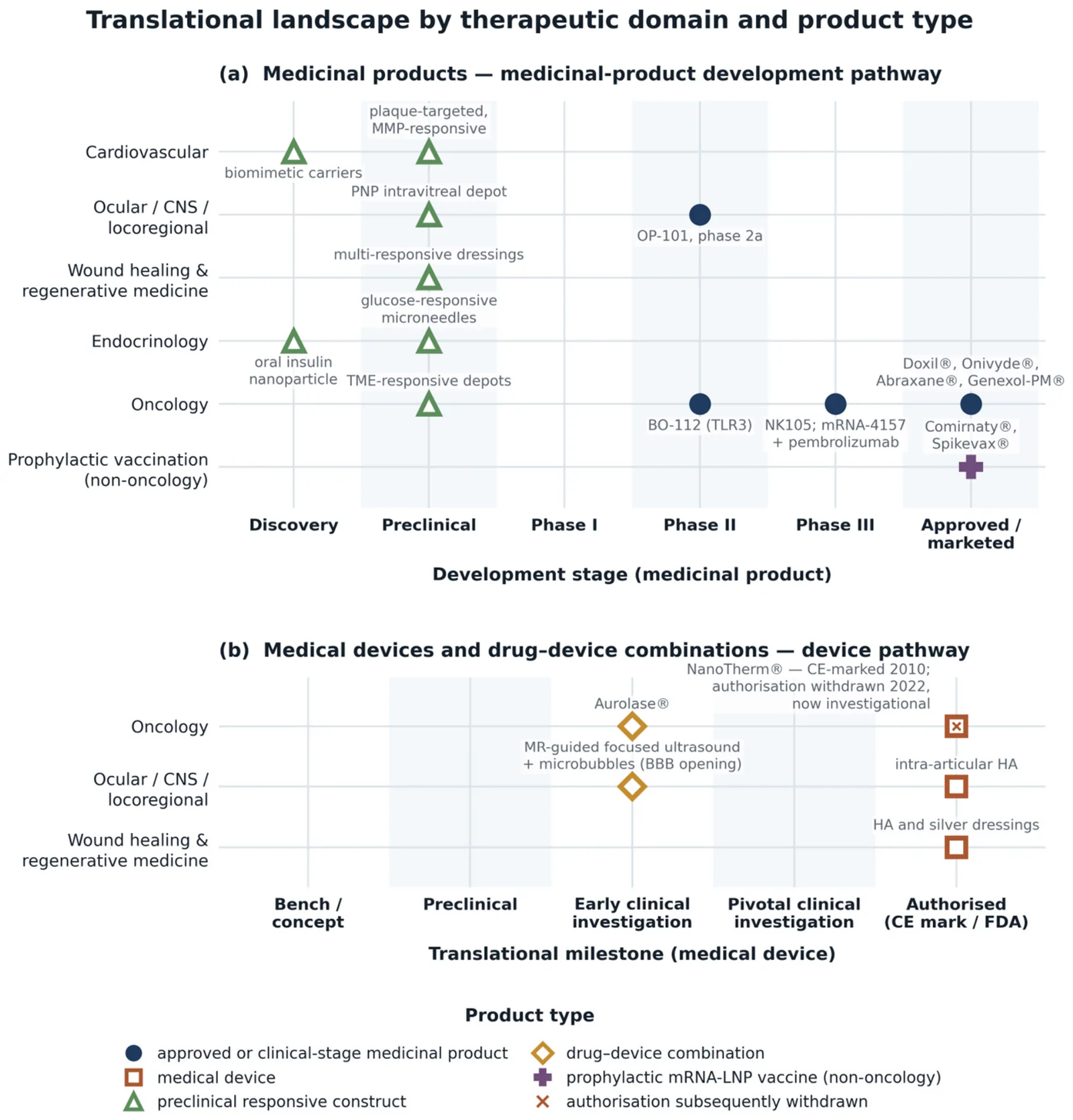
Translational landscape by therapeutic domain and product type, presented as two panels with separate translational axes. Panel (**a**) plots medicinal products and preclinical responsive constructs against the medicinal product development pathway, from discovery to marketing authorization. Panel (**b**) plots medical devices and drug–device combinations against device-specific milestones: bench and concept, preclinical, early clinical investigation, pivotal clinical investigation, and CE marking or equivalent authorization. The two are shown separately because the medicinal product and device pathways are not positions on a single developmental continuum and because Phase I–III designations do not apply to devices. All markers are drawn at a single uniform size; no quantitative encoding of publication activity is implied. Within each panel, marker position denotes the highest translational milestone reached by that entry, not the strength of its efficacy evidence. NanoTherm^®^ is accordingly placed at CE marking, the highest milestone it reached, and is flagged with a distinct symbol indicating that the authorization was subsequently withdrawn; the system is currently investigational and holds no marketing authorization in any jurisdiction [121]. The prophylactic vaccination row is included for consistency with Table 10, where it illustrates GMP nanomedicine manufacture at scale rather than a controlled-release application. Original figure created by the authors using Matplotlib 3.10.8 (Python 3.12.3).

The materials and mechanisms described in Section 3, Section 4, Section 5 and Section 6 are not abstract; they are deployed against specific clinical problems where conventional dosing has reached its limits. This section maps the four therapeutic domains in which nanoparticulate, hydrogel and hybrid carriers have advanced furthest: oncology, endocrinology (diabetes), wound healing/infection/regenerative medicine, and locoregional delivery (ocular, intra-articular, and central nervous systems) (Figure 8; Table 10).

### 7.1. Oncology

Cancer remains the dominant therapeutic indication for nanomedicine, with the majority of clinical-stage drug delivery formulations directed at solid-tumor and hematological malignancies [4,7,9,34,141]. The first generation of approved liposomal anthracyclines (Doxil^®^, Myocet^®^, and DaunoXome^®^), liposomal irinotecan (Onivyde^®^), liposomal vincristine (Marqibo^®^), albumin-bound paclitaxel (Abraxane^®^) and the polymeric micellar paclitaxel formulations (Genexol-PM^®^ and Nanoxel^®^, with NK105 in Phase III) was driven primarily by improved tolerability rather than transformative efficacy [7,61,62]. Contemporary effort has, therefore, shifted toward two complementary directions: tumor-microenvironment-responsive carriers that exploit acidic pH, elevated GSH, ROS and overexpressed enzymes to localize cytotoxic and immunomodulatory payloads [4,5,8,34,106], and chemo–immunotherapy combinations, in which nanocarriers co-deliver a chemotherapeutic and an innate immune agonist (TLR3, TLR7/8, STING, and RIG-I) to convert “cold” tumors into “hot” ones [127,141,142]. A 2025 *Angewandte Chemie* minireview cataloged over a dozen innate immune modulator nanoformulations now in clinical evaluation, including BO-112 (a TLR3 agonist polyplex), which produced immune activation in ≥46% of melanoma patients in Phase I and is now in Phase II combination trials [127]. Magnetic hyperthermia (NanoTherm^®^, CE-marked as a device in 2010 for recurrent glioblastoma (that marking since been discontinued), as detailed in Section 6.7) and plasmonic photothermal therapy (Aurolase^®^, in Phase II for prostate cancer) extend the toolkit to physical-trigger-based treatment [23,117,121]. mRNA cancer vaccines delivered by lipid nanoparticles have entered the clinical mainstream: in the randomized KEYNOTE-942/mRNA-4157-P201 Phase IIb readout, the personalized mRNA-LNP cancer vaccine mRNA-4157 (V940) combined with pembrolizumab improved recurrence-free survival relative to pembrolizumab alone in resected stage III/IV melanoma (with a hazard ratio of approximately 0.56, i.e., an approximately 44% relative reduction in the risk of recurrence or death over the trial follow-up); the agent, now designated intismeran autogene, has since advanced to a Phase III confirmatory trial in resected high-risk stage II–IV melanoma (INTerpath-001, NCT05933577), with a further Phase III trial running in non-small-cell lung cancer (NCT06077760) and additional mRNA vaccine trials active across pancreatic, glioblastoma and other indications [125,126]. The Phase 2b efficacy dataset and the current Phase III development stage are reported separately in Table 10, as they are distinct claims. Injectable nanocomposite hydrogels for intratumoral delivery of immune agonists, ICB-loaded NPs and mRNA are emerging as the next frontier, with several systems showing months-long depot release and complete tumor regression in preclinical solid-tumor models [11,15,34].

### 7.2. Endocrinology: Closed-Loop Insulin Delivery

The “artificial pancreas” pharmaceutical—a self-regulating system that releases insulin in proportion to ambient glucose—has been pursued for more than three decades. The mechanistic toolkit (Section 6.8) now includes glucose oxidase/pH-responsive matrices, phenylboronic acid (PBA)–diol crosslinked hydrogels, and concanavalin-A glucose-sensing networks [21,31,32,119]. Recent translational milestones include glucose-responsive microneedle patches (Gu and colleagues), which delivered insulin in proportion to interstitial glucose for ≥10 h in diabetic mice while avoiding hypoglycemia, and an oral glucose-responsive insulin nanoparticle that achieved subcutaneous-comparable glucose control in a type 1 diabetes model after a single oral dose [119]. A 2025 *Int J Pharm* review by Jiang and colleagues mapped more than 20 contemporary smart oral insulin platforms, identifying gastric stability, intestinal permeability and dose reproducibility as the principal translational hurdles [101]. PBA-based responsive networks have proved particularly versatile: incorporating PBA into PNP injectable hydrogels yields a single platform that responds simultaneously to glucose and ROS, the two principal pathological cues in poorly controlled diabetes [11,21,32].

### 7.3. Wound Healing, Infection and Regenerative Medicine

Chronic and infected wounds—particularly diabetic foot ulcers, which carry a 15–25% lifetime risk in patients with diabetes and remain a major cause of amputation—have become a flagship indication for multifunctional nanocomposite hydrogels [11,15,143,144]. The contemporary design philosophy, exemplified by the 2024–2025 literature, integrates four functionalities into a single dressing: antimicrobial action (silver, copper, and zinc oxide NPs; quaternized chitosan; antimicrobial peptides; and photothermal NIR sterilization), ROS scavenging (cerium-oxide, manganese-oxide, and polyphenol-loaded NPs), pro-angiogenic signaling (VEGF, PDGF, dihydromyricetin, and exosomes from MSCs), and immune modulation (M1→M2 macrophage polarization via TGF-β3, quercetin or natural flavonoids) [128,129,130,143,144]. A 2024 *Adv Sci* multi-responsive hydrogel by Dai et al. combined pH/ROS/glucose responsiveness, photothermal antibacterial action and angiogenic stimulation in a single material that closed methicillin-resistant *S. aureus*-infected diabetic wounds within 14 days in murine models [128]. A 2025 sprayable exosome-loaded hydrogel achieved sustained release with controlled spatial coverage [129]. A 2025 catechol-modified quaternized chitosan/PEG hydrogel co-delivered TGF-β3 with intrinsic antibacterial activity, accelerating wound closure 1.6-fold versus controls in diabetic models [130]. Granular and microgel-assembled hydrogels are simultaneously enabling cell-instructive injectable scaffolds for regenerative applications in cartilage, bone and cardiac muscle, where their interconnected porosity supports endogenous cell infiltration [11,17,30,95].

### 7.4. Ocular, Central Nervous System and Locoregional Delivery

The eye and the brain epitomize the challenge of delivering drugs to anatomically privileged compartments. Ocular delivery is constrained by rapid tear turnover (precorneal half-life of seconds to minutes), tight blood–aqueous and blood–retinal barriers, and the limited frequency at which intravitreal injections can be safely repeated (typically every 4–8 weeks for current anti-VEGF biologics) [33,58]. Polymer–nanoparticle (PNP) injectable hydrogels are among the more promising responses. A single intravitreal injection of a shear-thinning HPMC-C12/PEG-PLA NP hydrogel loaded with bimatoprost formed a slowly eroding depot in rabbits, raising the effective intraocular half-life of the drug from an estimated 0.2 to 1.7 days and leaving it measurable in the vitreous at eight weeks—the terminal time point of the study—from one administration [33]. The same study, however, reported a mild foreign-body response comprising foamy macrophages, multinucleated giant cells and minimal patchy fibroplasia; its authors note that no injectable hydrogel depot has yet been approved for ocular use, and that tolerability in the vitreous, rather than release duration, is the limiting problem [33]. Solid-lipid nanoparticles and nanostructured lipid carriers extend this approach to the anterior segment via topical administration [58]. Central nervous system delivery is constrained by the blood–brain barrier (BBB), which excludes 98% of small molecules and effectively all biologics. Strategies now in clinical evaluation include MR-guided focused ultrasound (FUS) with circulating microbubbles, which transiently and locally opens the BBB at sub-millimeter precision and has been used to deliver chemotherapy and biologics to glioblastoma and to demonstrate proof-of-concept in Alzheimer’s disease [42]. PAMAM-N-acetylcysteine dendrimers (OP-101, which has completed a randomized Phase 2a study, NCT04458298; Table 11) and biomimetic exosome-mimetic nanocarriers add targeted intracellular delivery to inflamed CNS regions [64,65]. Intra-articular delivery of NSAIDs, biologics and gene therapies via injectable hydrogels (HA-based, PNP, and granular) extends synovial residence from hours to weeks–months, with several formulations in late-stage clinical evaluation for osteoarthritis [11,17]. Other locoregional applications—intratumoral depots for cancer immunotherapy (Section 7.1), peri-anastomotic dressings, intrapericardial/myocardial injectables for ischemic heart disease [113], intra-bladder hydrogel formulations, and intra-articular anti-rheumatic delivery—round out the locoregional pipeline.

### 7.5. Cardiovascular Delivery

Cardiovascular disease remains the leading global cause of death, and the delivery problem it poses is distinctive: the target is a focal, chronically inflamed region of a vessel wall or myocardium embedded in a compartment through which the entire cardiac output passes every minute. Systemic anti-inflammatory and lipid-lowering therapy leaves a substantial residual risk, and the doses required to modulate plaque biology directly are limited by systemic immunosuppression [145,146]. Nanoparticulate and hydrogel carriers have accordingly been developed against five related targets—vascular inflammation, atherosclerotic plaque, acute and procedure-related injury to the vessel wall, thrombosis, and post-infarction myocardial remodeling—and the field has matured sufficiently in the past two years to warrant treatment as a distinct application domain rather than as an isolated cardiac example.

Regarding plaque and vascular inflammation, the pathological cues available in an atherosclerotic lesion map closely onto the sensing chemistries in Section 6: locally elevated reactive oxygen species, an acidic pH in the necrotic core, overexpressed matrix metalloproteinases and hyaluronidase in the fibrous cap, and an activated endothelium presenting adhesion molecules that are absent from quiescent vessel walls [146,147]. Biomimetic carriers exploit the last of these directly: monocyte-membrane-coated nanoparticles inherit the adhesion repertoire of the cell they are derived from, accumulate preferentially in the intima of atheroprone arteries while evading phagocytes, and have been used to deliver verteporfin so as to suppress YAP/TAZ signaling specifically in inflamed endothelium, reducing plaque development without histopathological change in major organs, an outcome that systemic YAP/TAZ modulation could not achieve safely [133]. Macrophage-microvesicle-coated, carrier-free co-assemblies of ginsenoside Rb1 and probucol pursue the same targeting principle with a different payload logic, simultaneously scavenging intracellular ROS, suppressing pro-inflammatory cytokine secretion and inhibiting lipid deposition [148].

Stimuli-responsive release has been demonstrated in the same setting. Hyaluronidase-responsive supramolecular nanoassemblies incorporating a silver sulfide core disassemble selectively in the enzyme-rich plaque microenvironment, releasing aspirin and lovastatin in a programmed sequence with cumulative release exceeding 80% over 72 h, while second-window near-infrared photothermal activation both accelerates release and exerts a local anti-inflammatory effect; the plaque area fell by 53% in apolipoprotein-E-deficient mice, with restored elastic-fiber integrity and increased collagen deposition [134]. A multifunctional construct combining PLGA nanoparticles within a zinc–epigallocatechin gallate metal–organic framework shell delivers a CpG oligonucleotide to plaque macrophages and addresses plaque clearance through a defined mechanistic cascade, restoring efferocytosis of apoptotic foam cells, promoting intracellular lipid degradation and upregulating cholesterol-efflux transporters [149]. Dual-targeting strategies that combine a ligand for a cellular or subcellular address with a stimulus-responsive release element represent the current design consensus and have been reviewed critically with attention to the limitations of single-targeting approaches [150]. A protein-assembled construct gives that consensus a concrete instance and supplies the second quantitative in vivo result the subsection otherwise lacked. Epigallocatechin-3-gallate and protamine were co-assembled non-covalently, and the resulting particles were surface-coated with fucoidan, a single ligand that addresses two cell populations at once, P-selectin on the inflamed endothelium and the class A scavenger receptor on foamy macrophages—while the arginine-rich protamine core doubles as a nitric oxide source [135]. Two cues act on separable elements of the construct: trypsin, used as a surrogate for the ectopic proteases of the plaque, degrades the protamine core and the fucoidan shell, and hydrogen peroxide reacts with the guanidinium groups of protamine to generate nitric oxide. Measured by dialysis at 37 °C, half of the encapsulated epigallocatechin gallate was released in approximately 6.4 h at pH 6.8 in the presence of trypsin, against 29.9% cumulative release at 24 h in its absence, and nitric oxide release was sustained above 3 µM for 60 h in 100 µM of hydrogen peroxide, with the untriggered controls reported under the same conditions. The logic relating the two cues is not established, since the four conditions required by Section 6.9 are not reported under a common assay. In apolipoprotein-E-deficient mice on a high-fat diet, dosed intravenously twice weekly for eight weeks at 3.12 mg of epigallocatechin gallate and 7.81 mg of fucoidan per kilogram, aortic lipid accumulation fell approximately 11.3-fold relative to the untreated high-fat-diet control and approximately 2.4-fold relative to the same particles coated with polyacrylic acid and administered together with free fucoidan, an internal comparator that separates the contribution of the targeting shell from that of the payload [135].

This section discusses injury to the vessel wall, including restenosis and dissection. These two settings, absent from the plaque literature, extend the same design logic to acute and procedure-related vascular injury. After balloon angioplasty, the denuded intima exposes type IV collagen, while the local reactive oxygen species concentration rises, and both cues can be used at once. A hydroxypropyl-β-cyclodextrin scaffold bearing an ethyl-caffeate phenylboronic ester was loaded with isosorbide dinitrate by host–guest inclusion and decorated with a collagen-IV-homing peptide, so that the oxidative environment that triggers hydrolysis of the boronate is also consumed by it and by the 4-hydroxybenzyl alcohol released as a by-product [136]. In dialysis at 37 °C, cumulative release reached 96.13 ± 0.06% over 24 h in 1 mM hydrogen peroxide against a substantially lower plateau in phosphate-buffered saline measured in parallel; because the profile rests on a dialysis method alone, it is reported here as an observed profile rather than an identified mechanism, per Section 2.3. In balloon-injured rat carotid arteries dosed intravenously on days 0, 3, 6, 9 and 12 and assessed on day 14, the targeted dual-function construct produced the largest reduction in intimal area and the proliferation index across six arms, whereas the nitric oxide-only arm did not relieve intimal thickening at all, and in individual animals, it produced worse results than injury alone [136].

Aortic dissection has been approached through a target expressed across all three implicated cell types rather than through a single-cell address. Galectin-3 is upregulated on the inflamed endothelium, macrophages and smooth-muscle cells as dissection progresses, and modified citrus pectin both binds and inhibits it, so that the targeting ligand is itself an active agent; co-assembled with protamine and a carboxymethyl-cyclodextrin–resveratrol inclusion complex, the resulting particles respond to acidic pH, protease and hydrogen peroxide, with nitric oxide generation characterized at 100 µM of hydrogen peroxide, and release resveratrol and nitric oxide sequentially rather than together, a cascade in the sense of Figure 7 rather than a gate [137]. In β-aminopropionitrile/angiotensin-II-induced dissection, aortic accumulation rose from 2.66-fold over healthy controls in week 1 to approximately 20-fold in week 4, tracking the galectin-3 expression profile; twice-weekly intravenous treatment over four weeks reduced the incidence of dissection from 87.5% to below 25% and raised four-week survival from 15% to 88% (n = 8 per group) [137]. This is the largest effect size in the cardiovascular material assembled here, and its source is worth naming: not a more sophisticated trigger, but a target that appears early and in every cell type that matters.

The other principal aortic indication, abdominal aortic aneurysm, illustrates a different point, because here, the released species is itself an antioxidant. Benzylthiol-modified hyperbranched polylysine (with a degree of substitution of 85.4%) was self-assembled with selenomethionine into core–shell particles of 40–70 nm carrying 25.4 mg of selenomethionine per gram, in which oxidation of the phenylthio groups to the corresponding sulfoxides drives a hydrophobic-to-hydrophilic transition and liberates the cargo [138]. The rationale for encapsulating selenium at all is a therapeutic window argument rather than a targeting one: selenium is required for glutathione peroxidase 4 activity, but the interval between its effective and its toxic dose is narrow, which is precisely the problem a controlled-release carrier exists to solve. In calcium-chloride-induced aneurysm in mice, a single intravenous dose administered four weeks after induction reduced aortic diameter, wall thickening and collagen deposition relative to both the untreated model and the drug-free carrier, and restored glutathione peroxidase 4 and SLC7A11 and FTH1 expression while raising superoxide dismutase and glutathione and lowering malondialdehyde [138]. Two features of the evidence deserve comment under the conventions of Section 2.1 and Section 2.3. The drug-free carrier arm was essentially inactive, which attributes the effect to the selenomethionine rather than to the particle, but no free-selenomethionine comparator was included, so this study does not establish that the formulation outperforms the unencapsulated drug, the comparison that Section 7.6 argues should accompany every such claim. And the responsiveness is reported as a comparison between 1 and 10 mM of hydrogen peroxide, without an untriggered control under identical conditions; according to the criterion in Table 2, this demonstrates concentration dependence rather than a characterized on/off contrast, and both concentrations sit two orders of magnitude above the lesion levels in Table 9.

After myocardial infarction, the delivery problem changes from chronic modulation to timed intervention in an evolving inflammatory sequence, in which early neutrophil and macrophage responses clear necrotic tissue, but sustained activation drives adverse remodeling [151]. Carriers here are used less to accumulate passively than to time an intervention. Matrix-metalloproteinase-responsive peptide–polymer amphiphile micelles aggregate within infarcted myocardium after intravenous administration and are retained for up to 28 days, converting an enzymatic signature of injury into localization and prolonged residence [113]. Nanoparticle systems directed at neutrophil phenotype switching, macrophage polarization and regulatory T-cell balance have been assembled around pH-, ROS- and enzyme-responsive release, with targeting precision, manufacturing scalability and long-term biosafety identified as the outstanding obstacles [151]. Injectable hydrogel depots for intramyocardial and intrapericardial administration extend the same logic to a locoregional route. A peptide–drug conjugate rather than a nanoparticle formulation makes a further and different point. Isosorbide mononitrate was joined to a cardiac-injury-homing cyclic peptide through a phenylboronic ester that undergoes 1,6-elimination on reaction with hydrogen peroxide, releasing the nitrate without residue and liberating 4-hydroxybenzyl alcohol, itself an antioxidant, as the by-product [139]. The conjugate is a single molecule of a defined structure that self-assembles into 47 ± 4 nm aggregates, and its authors argue explicitly that this confers batch-to-batch control that multi-component nanoformulations cannot match, a manufacturing argument of the kind Section 8 identifies as decisive, rather than a material one. Cumulative release reached 86.0 ± 3.0% at 24 h in 1 mM of hydrogen peroxide against less than 15% in phosphate-buffered saline over the same period, with only a marginally elevated profile at 10 µM. In mice after left-anterior-descending coronary ligation, dosed intravenously every three days for four weeks at 200 µg/kg isosorbide mononitrate equivalent, the conjugate improved ejection fraction and fractional shortening at 28 days and reduced the fibrotic area and cardiomyocyte cross-sectional area, while the scrambled peptide conjugate achieved only partial recovery and the free nitrate essentially none [139].

Thrombosis was named above as one of the five targets and is the least developed of them, in part, because the therapeutic window for fibrinolysis is narrow, and the penalty for releasing a thrombolytic in the wrong place is hemorrhage. One construct addresses both halves of that problem with the same chemistry. Fucoidan and gelatin were assembled with iron oxide nanoparticles so that the gelatin restricts water-proton access, suppressing the T1-weighted signal while amplifying T2. Matrix metalloproteinase-2 and -9 within the thrombus disassemble the construct and invert the contrast, and the resulting T1/T2 ratio varies with thrombus age (logarithmic fit, R^2^ = 0.911) and with collagen density [140]. The same disassembly releases urokinase—approximately 90% within 2 h in the presence of 0.75 µg/mL of matrix metalloproteinase-9 in phosphate-buffered saline at pH 6.8 and 37 °C, against 10–15% in its absence under identical conditions, which is among the cleaner on/off comparisons in the literature according to the criterion in Table 2. Two boundaries apply when reading it. The imaging function is a diagnostic capability and not controlled cargo release and is not counted as such here (Section 2.1), and the thrombolytic result is an in vitro clot lysis measurement, so the reduction in hemorrhagic risk remains a property of the release mechanism rather than a demonstrated in vivo outcome.

One result recurs across these systems and deserves to be stated separately, because it is a point about release rather than about cardiology. Wherever a control arm received the same nitric oxide donor without the accompanying antioxidant chemistry, that arm produced inert or worse results than no treatment at all. Intimal thickening was not relieved, and in individual animals, it progressed further [136], and intracellular peroxynitrite and myocyte loss rose above that in the untreated control [139]. A third system co-locates an antioxidant with the nitric oxide source for precisely this reason [135]. When the cue is a reactive species, trigger and payload are not independent. A carrier that senses reactive oxygen species and simultaneously depletes them protects its own cargo from the environment that released it, and the therapeutic effect is attributable to that coupling rather than to delivery of the drug as such. This is the oxidative–reductive paradox of Section 6.4 in its practical form, and it is a case in which the released species framework of Section 2.5 does real work: the species released is nitric oxide, but what determines the outcome is what else is present when it arrives.

Read against the framework of Figure 2, cardiovascular nanomedicine is instructive precisely because it is less clinically advanced than oncology. The sensing chemistries are the same ones that have reached patients in oncology. The carrier architectures are the same; what differs is the administration route and the risk tolerance of the indication. A chronic, largely asymptomatic, effectively treatable disease sets a far higher safety bar for a systemically administered nanocarrier than metastatic malignancy does, and this—not any deficiency of trigger chemistry—is the principal reason no stimuli-responsive cardiovascular carrier has entered late-phase clinical evaluation. This is a clear instance of the general pattern developed in Section 8: clinical readiness tracks route and indication risk rather than materials sophistication. The six primary systems added to this subsection are consistent with that reading and sharpen it in three ways. All six are preclinical rodent studies with no registered clinical evaluation, so the claim of the preceding sentence survives contact with the most recent primary literature rather than merely predating it. Second, hydrogen peroxide is one of the cues in five of the six systems, and in three of those five, the reported characterization is at or above 1 mM—1 mM [136], 1 and 10 mM [138], and 1 mM against a profile only marginally elevated at 10 µM [139]. That is an order of magnitude or more above the concentrations reported in inflamed tissue, and it places those three squarely in the gap between trigger contrast and clinically achievable activation set out in Table 9. The remaining two report hydrogen peroxide-triggered nitric oxide generation at 100 µM [135,137], and the sixth system is matrix metalloproteinase-responsive rather than oxidatively triggered [140]. The limitation is, therefore, quantitative rather than conceptual, and it is not uniform across the subsection. Third, and against the general pattern, the peptide–drug conjugate answers the manufacturing objection rather than the chemical one, by replacing a multi-component assembly with a single molecule of a defined structure [139]. If cardiovascular delivery does close the distance on oncology, the evidence assembled here suggests it will be through a move of that kind—reproducibility and route—rather than through a new trigger.

### 7.6. Two Worked Comparisons Against Conventional Formulations

Reviews of this field commonly report what a carrier achieves without stating what the same drug achieves without it. Two cases are set out here in which the comparison against the conventional formulation is available, one from approved clinical practice and one preclinical, chosen because they illustrate opposite lessons.

Case 1—liposomal irinotecan versus conventional irinotecan: Irinotecan is a camptothecin prodrug whose active metabolite SN-38 has a narrow therapeutic index; conventional intravenous administration produces rapid clearance and dose-limiting neutropenia and diarrhea. Encapsulation in a PEGylated liposome with an intraliposomal sucrose octasulfate gradient converts short-lived systemic exposure into a depot that circulates and converts to SN-38 over a prolonged interval [7]. In metastatic pancreatic adenocarcinoma after gemcitabine failure—a setting in which conventional irinotecan had no established benefit—the liposomal formulation combined with 5-fluorouracil and folinic acid produced an overall survival advantage over the fluoropyrimidine control, and it is on that basis that the product holds marketing authorization [7]. The instructive point is what the improvement is attributable to. The carrier is not stimuli-responsive and not actively targeted; the benefit derives from altered pharmacokinetics and the resulting change in the tolerable dose intensity. This is the pattern across the approved nanomedicines: improved tolerability rather than transformed potency, achieved by controlling when the drug is available rather than where [142,152].

Case 2—a polymer–nanoparticle intravitreal depot versus topical dosing: Bimatoprost is administered as daily drops, and the precorneal half-life of an ophthalmic solution is measured in seconds to minutes, so that adherence dominates real-world effectiveness. A single intravitreal injection of a shear-thinning hydroxypropyl-methylcellulose-C12/PEG-PLA polymer–nanoparticle hydrogel loaded with bimatoprost formed a slowly eroding depot in rabbits, raising the effective intraocular half-life of the drug from an estimated 0.2 to 1.7 days and leaving it measurable in the vitreous at eight weeks—the terminal time point of the study—from one administration [33]. Against a daily-drop comparator, this is a change in kind rather than degree. The same study, however, reported a mild foreign-body response comprising foamy macrophages, multinucleated giant cells and minimal patchy fibroplasia, and its authors note that no injectable hydrogel depot has yet been approved for ocular use [33]. The limiting variable is vitreous tolerability, not release duration, which is the second half of the lesson. Demonstrating superiority on the pharmacokinetic endpoint does not by itself establish a translatable advantage, and the endpoint on which these systems fail is rarely the one on which they are optimized.

Only trials for which the registry entry was individually verified are listed. This table is not an exhaustive census of nanomedicine trials; the inclusion criterion is that the agent is discussed in the text of this review and that a registry identifier could be confirmed. Entries for which registry verification remained incomplete at the time of writing have been omitted rather than reported from secondary sources.

## 8. Translational Considerations

The gap between a paper-published “successful” formulation and a clinically deployed pharmaceutical remains the single greatest obstacle in the field. A 2024–2025 industry perspective in the *Journal of Controlled Release* notes that, despite more than 15 FDA-approved cancer nanomedicines and a robust clinical pipeline, the majority of nanocarrier programs still stall before Phase II, for reasons that recur with striking consistency across mechanism classes [152]. This section synthesizes the principal translational hurdles into four clusters: chemistry, manufacturing, and controls (CMC), regulatory frameworks, immunogenicity and biological barriers, and the structural features of the contemporary clinical trial landscape.

**Chemistry, manufacturing and controls (CMC):** Nanoparticulate and hydrogel systems are typically classified as “complex drug products” or, in the EMA framework, “non-biological complex drugs” (NBCDs); their structural and functional integrity depend on dozens of process variables (lipid composition, mixing geometry, mixing rate, temperature, ionic strength, and sterilization route) that classical small-molecule release profile testing cannot fully capture [152,153,154,155]. Batch-to-batch variability in particle size, polydispersity, surface chemistry and drug load translates directly into pharmacokinetic and pharmacodynamic variability. The advent of microfluidic mixing, continuous manufacturing and “quality-by-design” frameworks has begun to mitigate these concerns—and the rapid scale-up of mRNA-LNP vaccines during the COVID-19 pandemic demonstrated that GMP nanomedicine manufacturing at billions of doses per year is feasible—but extending these advances to multi-component hybrid hydrogel–nanoparticle systems, microgels and biomimetic membrane-coated NPs remains a substantial engineering challenge [152,155].

**Regulatory frameworks:** Nanomedicines do not fit neatly into the small-molecule or biologics pathways for which the FDA, EMA and PMDA were originally designed [153,154]. A 2025 *Small* review by Desai et al. consolidated current global approval trends and noted that case-by-case review remains the norm, with each agency applying nanotechnology-specific reflection papers and guidance documents (FDA Drug Products Containing Nanomaterials Guidance, 2022; EMA Reflection Paper on Nanomedicines; ICH Q9R1 Quality Risk Management) on top of standard CTD modules [154,156]. Particular complications arise for hybrid biological–synthetic systems (cell-membrane-coated NPs and genetically engineered exosomes), for stimuli-responsive products requiring concomitant device approval (FUS systems for ultrasound triggering and MR-coupled magnetic actuators), and for personalized formulations (mRNA cancer vaccines), each of which strains the boundaries of the existing classification. Recent calls for harmonization across jurisdictions, for nanomedicine-specific characterization standards, and for adaptive trial designs have begun to converge in regulatory editorials [154,156,157].

**Immunogenicity and biological barriers:** A widely under-appreciated barrier is the immunogenicity of PEG itself. A landmark 2025 *Nature Reviews Bioengineering* review by Chen, Su and Roffler consolidated the evidence that anti-PEG antibodies—induced by repeated administration of PEGylated medicines and now also detectable in 50–90% of healthy adults owing to ubiquitous PEG exposure in cosmetics and food additives—accelerate blood clearance, alter biodistribution, and underpin most rare but serious hypersensitivity reactions to PEGylated formulations and mRNA-LNP vaccines [123,124]. Several 2025 strategies are now in active development to mitigate this: hydroxy-terminated PEG, randomized PEG (rPEG) constitutional isomers, sulfur-ylide-terminated PEG, and PEG alternatives such as polysarcosine, poly(2-oxazoline)s and zwitterionic polymers [123,158,159]. Protein corona dynamics, which modify the effective surface chemistry of every NP within seconds of plasma contact, similarly determine biodistribution and complement activation in ways still incompletely predictable from in vitro characterization [152,155]. Mononuclear phagocyte clearance continues to divert most of the injected dose of systemic nanomedicines to the reticuloendothelial system, with biodistribution studies routinely reporting 50–80% accumulation in the liver and spleen; this remains the largest contributor to the gap between rodent and human EPR-mediated tumor accumulation [9,10,83,152]. The Wilhelm meta-analysis (Section 3 above) and its updates [41,81,82] are best read in this context.

**Clinical trial landscape:** Across all therapeutic areas, the mismatch between preclinical promise and clinical efficacy is structural rather than coincidental. Rodent tumor models routinely overstate the EPR effect; tight inclusion criteria limit recruitment to small populations; and the high heterogeneity of patient tumor biology exposes the limits of one-size-fits-all formulations [9,10,83,152]. Several recent meta-analyses of nanoparticle randomized controlled trials in oncology found that improved tolerability, rather than improved overall survival, was the most reproducible benefit relative to standard-of-care chemotherapy [142,152]. Bridging this gap requires three concurrent shifts: integration of patient-specific multi-omics data into formulation choice (so that the carrier is matched to the patient, not vice-versa); modular, quality-by-design manufacturing platforms that allow rapid swap-in of different cargoes onto the same regulatory dossier; and adaptive trial designs that can re-select responder populations as biomarkers emerge [152,157].

Route and product type predict readiness better than chemistry. Setting the entries of Table 10 and Figure 8 against one another produces a pattern that the carrier-type organization of the preceding sections conceals. The formulations that have reached marketing authorization are almost entirely intravenous, non-responsive and regulated as medicinal products—liposomal anthracyclines and irinotecan, albumin-bound paclitaxel, polymeric micellar taxanes, and ionizable-lipid nucleic acid carriers. Products employing an exogenous physical trigger have reached patients only through the medical device pathway, and their subsequent history has been governed by commercial and reimbursement factors rather than by their release physics. NanoTherm was CE-marked as a device in 2010 for recurrent glioblastoma, but following the sponsor’s insolvency in 2022, the technology no longer holds an active marketing authorization, and its current developer states that the prior CE marking has been discontinued, and the system is investigational [23,117,121]. Meanwhile the entire class of endogenously triggered, stimuli-responsive carriers—the chemistry that occupies most of Section 6 and most of the recent literature—remains preclinical or in early-phase evaluation across every therapeutic domain in Figure 8.

The inference is not that stimuli responsiveness fails, but that it is not the variable on which translation currently depends. Three factors order the entries better. The first is administration route: locoregional administration removes at a stroke the biodistribution problem that the Wilhelm analysis quantifies, which is why intravitreal, intra-articular and intratumoral depots advance on the strength of local tolerability data rather than of targeting efficiency [33,81,152]. The second is regulatory product type, which determines the evidentiary burden before it determines anything else; a device pathway and a medicinal product pathway are not points on one developmental continuum, and plotting them as though they are overstates the maturity of the field, which is why Figure 8 presents the two pathways as separate panels. The third is the risk tolerance of the indication, which is why identical sensing chemistries sit at Phase III in oncology and remain preclinical in cardiovascular disease (Section 7.5). A carrier program is, therefore, better assessed by asking which route it can use, which regulatory class it falls into, and what safety margin its indication permits, than by asking how selective its trigger chemistry is.

These obstacles are common to complex carriers of every kind. A second set is specific to the trigger chosen and is compared across the ten stimulus classes in Table 9 at the close in Section 6. Two of those stimulus-specific liabilities bear directly on the translational discussion above. The first is that the activation thresholds demonstrated in vitro frequently exceed those achievable in patients, most clearly for glutathione, where characterization at 50–100 mM against a physiological intracellular range of 1–10 mM inflates the apparent on/off contrast of a whole class of carriers [106]. The second is that exogenously triggered systems import the regulatory burden of a device alongside that of a medicine, which is the mechanism behind the product-type pattern described above.

Intellectual property and marketed products: Reviews of this field seldom examine the patent position, although it constrains translation as directly as biology does. Table 12 summarizes representative granted patents and marketed products across the platform classes. Three observations are worth drawing out. The foundational composition-of-matter patents on the earliest platforms—PEGylated liposomes, albumin-bound paclitaxel, and the core PLGA depot technologies—have expired, which is why generic and hybrid applications now exist for several of these products and why the regulatory discussion has shifted toward demonstrating equivalence for complex products, a problem that returns directly to the release-testing methodology in Section 2.3 [37,39,40]. Ionizable lipid chemistry, in contrast, is densely and contentiously patented, and the freedom-to-operate position rather than the formulation science has been the limiting factor for several mRNA programs. Finally, stimuli-responsive hydrogel and hybrid constructs are patented overwhelmingly as platform or method claims rather than as products, which is consistent with their preclinical status and means that the commercial value currently resides in the delivery method rather than in any specific medicine.

Patent families in this field are large and jurisdiction-specific; this table characterizes the position at the platform level and is not a substitute for a freedom-to-operate analysis. The reference column cites the product and platform literature from which each entry is drawn; it is not a citation to the patent documents themselves, which were not individually retrieved.

## 9. Conclusions and Future Perspectives

The next decade of controlled-release research will be shaped by three convergent forces visible in the 2024–2026 literature and by one observation that cuts across all of them.

First, artificial intelligence-guided formulation is beginning to move from descriptive correlation towards prospective design, but the evidence for that transition is emerging rather than established, and it remains predominantly retrospective. Most of the 2024–2026 literature on the subject consists of reviews and perspectives that describe the direction of travel rather than primary studies that test it [35,36,50,160], and the primary work that does exist is trained on historical release datasets and evaluated by held-out cross-validation rather than by prospective experiments [51]. Prospective validation in the strict sense—a model-derived design criterion used to prepare a new formulation, whose measured release profile is then compared against the prediction—has so far been reported in only a small number of studies. The clearest is a tree ensemble (light gradient boosting) model trained on 181 release profiles from polymeric long-acting injectables and interpreted by Shapley additive explanation analysis; design criteria extracted from that analysis were used to prepare fast- and slow-releasing PLGA microparticle formulations of two drugs held out of the training set, and predicted and experimental profiles agreed closely, although the slow-release system diverged beyond day 15 as polymer hydrolysis altered the release rate, a failure mode lying outside the model’s feature space [49]. That is a genuine proof of principle, and it also delimits the following claim: a two-formulation confirmation within a single carrier class is not the same as demonstrated prospective design capability across nanoparticulate and hydrogel systems generally, and the stronger claim is not yet supported by the primary literature. A recent review documented end-to-end machine learning-empowered nanoparticulate design pipelines to compress formulation campaigns by an order of magnitude in time, although such gains are claimed for in silico screening rather than full GMP development [35]. The caveats are equally clear-eyed, and they connect directly to Section 2.3. The in vitro release datasets on which most models train are heterogeneous not only in the dissolution medium composition and sampling cadence but also in the release test method itself, and since dialysis and sample-and-separate techniques return materially different profiles from the same material [37,38,39], a model trained across methods is partly learning the assay rather than the formulation. Cross-laboratory generalization will remain fragile until a release-testing methodology is harmonized and reported, which is the practical case for the FAIR data infrastructure now being assembled [40,52]. Explainable models can identify dominant predictors (encapsulation efficiency, polymer molecular weight, and particle size) but degrade outside the training data manifold, and quality-by-design and design-of-experiments approaches retain real advantages where the mechanism is understood and the formulation space is low-dimensional [35,36,49,51,52]. The April 2025 FDA roadmap on reducing animal testing in preclinical safety studies, which announced acceptance of new approach methodologies, including organoids, organ-on-chip systems and validated computational models for selected biologic applications, is widely interpreted as an acceleration signal rather than the abolition of animal testing, and will further legitimize computational and organoid evidence in regulatory dossiers [161,162]. Patient-specific in silico representations, or theranostic digital twins, extend the same computational logic to individualized dose and release scheduling and are being adapted from radiopharmaceutical oncology to nanomedicine [163,164,165]; the concept is coherent, but the validation evidence connecting it to controlled-release design specifically remains thin, and it is noted here as a direction rather than an established capability.

Second, multi-stimuli AND-gated logic and biomimetic carriers will continue to sharpen the spatial selectivity of release while reducing off-target activation. The growth of cell-membrane-coated NPs, genetically engineered exosomes, DNA nanostructure-based responsive carriers, and supramolecular logic gates that combine endogenous and exogenous triggers points toward delivery vehicles that approach the discrimination of natural cellular signaling [25,26,34,70,71,72,106]. Hybrid hydrogel–nanoparticle constructs in particular offer a platform on which multi-stimuli logic can be engineered modularly.

Third, and most quietly, the field is beginning to confront sustainability, equity and reproducibility. Greener nanomanufacturing—solvent-free synthesis, plant-derived polymers, and low-energy processing—is a recurring theme in 2024–2025 regulatory editorials [157]. FAIR (findable, accessible, interoperable, reusable) data infrastructure for in vitro release datasets is being built to allow both AI training and reproducibility audits [52]. Sex-balanced preclinical studies and ethnically diverse clinical populations are emerging as prerequisites for translational claims that generalize beyond the historical defaults [152]. Equitable access—particularly to mRNA, gene therapy and theranostic formulations whose costs frequently exceed US$100,000 per patient course [125]—is increasingly recognized as a research priority, not merely a policy afterthought.

Underlying all four is the observation this review has been organized to expose. The field describes itself by carrier chemistry, and carrier chemistry turns out to be the wrong organizing variable for both of the questions practitioners most need answered. It does not predict release behavior because that is governed by the identity of the released species and the transport step limiting it—which is why a power-law exponent validated on dissolved drug misdescribes intact particle escape, and why a statistical fit obtained under an unreported assay geometry establishes very little at all. And it does not predict clinical readiness because that is governed by administration route, regulatory product type and the risk tolerance of the indication, which is why chemistries at Phase III in oncology remain preclinical in cardiovascular disease, and why the only physically triggered product to reach patients did so through a device pathway and has since lost its authorization. Neither point is visible from within a single material class; both follow from reading the nanoparticulate, hydrogel and hybrid literatures against one another. The practical recommendations are correspondingly concrete: state the released species, report the assay conditions, supply orthogonal evidence before claiming a mechanism, and assess a program’s translational prospects by its route and regulatory class before its trigger chemistry.

### Limitations of This Review

Four limitations should be kept in mind. First, as a narrative rather than systematic review, the literature selection reflects the authors’ subject-matter judgement, and some platforms and indications receive deeper coverage than others; topics summarized only briefly include exosome-based and extracellular vesicle delivery beyond their use as cargo, peptide self-assembly, RNA delivery beyond lipid nanoparticles, anterior-segment ocular delivery, and inhalation and nasal mucosal pulmonary delivery. Second, the breadth of scope retained here is a deliberate choice in service of the cross-material argument of Section 2.5, and it carries a real cost: a review confined to a single material class could treat that class in more depth than is possible here, and readers seeking an exhaustive treatment of any one platform should consult the focused reviews identified in Table 1. Third, the rapid pace of the field means any cut-off is partly arbitrary; this review prioritizes 2022–2026 work, and developments published during revision are likely to have appeared by publication. Fourth, several headline quantitative claims summarized here—nanoparticle delivery efficiency to tumors, clinical pipeline indications, and machine learning-driven time-to-formulation gains—derive from heterogeneous primary studies whose methodologies do not permit precise meta-analysis, and the release-testing heterogeneity documented in Section 2.3 means that release values compared across studies should be treated as indicative rather than as commensurable measurements; we have, therefore, presented these as orders of magnitude wherever the underlying evidence does not justify a sharper figure.

Taken together, the controlled release field has come a remarkable distance since the introduction of Doxil^®^ three decades ago. Nanoparticulate and hydrogel vehicles, individually and increasingly in hybrid constructs, are no longer aspirational platforms but the workhorses of a growing fraction of the global pharmaceutical pipeline. What has changed in the past five years is not only the materials toolkit but the design philosophy: from passive carriers chosen for tolerability to programmable, stimuli-responsive, patient-adapted systems chosen for the disease they treat and the patient who is treated. The challenges that remain—translational, regulatory, immunological, and ethical—are genuine but solvable, and the integration of mechanistic depth, AI-assisted design and disciplined translational science maps a credible path forward. The carrier, after all, is not the therapy; it is the means by which a therapy reaches its target. Realizing the full potential of stimuli-responsive controlled release is, in the end, a problem of converting our increasingly precise mechanistic understanding into therapies that more patients can actually receive.

## Figures and Tables

**Table 1:** Principal recent reviews covering this subject area, and the respect in which the present review differs.

Recent Review (Ref.)	Principal Coverage	Organizing Principle	What the Present Review Adds
Smart hydrogels for drug delivery [1,14]	Hydrogel chemistries and responsive mechanisms	By stimulus and polymer class	Places hydrogels against nanoparticulate and hybrid systems, and treats hydrogel release as one of four released-species cases rather than as a self-contained problem
Nanoparticle–hydrogel hybrid materials [11,15]	Hybrid architectures, coupling chemistries, and applications	By hybrid architecture	Adds the four-way released-species classification (Section 5.2), the matrix chemistry effect on release from embedded particles (Section 5.4), and the dissolved-drug baseline needed to show why exponent analysis fails for particulate release
Nanomaterial-integrated hydrogel composites [12]	Interfacial coupling, mechanical reinforcement, and swelling	By filler type and mechanical outcome	Carries the interfacial argument through to release-testing methodology and to the kinetic models that may legitimately be applied
Stimuli-responsive nanocarriers, general [4,6]	Carrier platforms and responsive chemistries	By carrier type and then stimulus	Distinguishes response modes (AND, OR, additive, sequential, or cascade) and states the experimental criteria for each (Section 6.9); separates controlled release from targeting and from barrier modulation (Section 2.1)
Tumor-microenvironment-responsive polymer platforms [34]	Endogenous triggers in oncology	By stimulus, within one indication	Extends the same sensing chemistries across four further therapeutic domains including cardiovascular (Section 7.5) and asks why identical chemistries sit at different stages in different indications
Translational record of nanomedicine [9,10]	Barriers to clinical translation	By barrier type	Separates regulatory product type from development stage (Table 10; Figure 8) and identifies route and indication risk as better predictors of readiness than trigger sophistication (Section 8)
Machine learning in formulation [35,36]	ML methods applied to release prediction	By algorithm and application	Connects the data quality bottleneck to the specific finding that the release test method changes the measured profile, so that models trained across methods partly learn the assay (Section 2.3 and Section 9)

**Table 2:** Definitions adopted throughout this review, and the criterion distinguishing each from its neighbors.

Term	Definition Adopted Here	Distinguishing Criterion	Common Conflation Avoided	Ref.
Sustained release	Prolongation of drug availability beyond that of an immediate-release form, without a defined target concentration window	Duration is extended; the rate is not specified	Treated as synonymous with controlled release, although a sustained profile may be highly non-uniform	[3,27]
Controlled release	Release engineered to maintain drug within a defined concentration window over a defined period	A target rate or profile is specified in advance and achieved	Claimed for any prolonged profile, including uncontrolled first-order decay	[3,27,29]
Stimuli-responsive release	Release rate altered by a defined endogenous or exogenous cue, with a demonstrated difference between the on- and off-states	Requires an off-state control under identical assay conditions	Claimed from the triggered condition alone, without the untriggered comparator	[4,5,6,34]
Targeted delivery	Preferential accumulation of the carrier at a site, by ligand–receptor recognition, physical guidance or biomimetic surface presentation	Alters where the carrier goes, not when cargo leaves it	Presented as controlled release; a targeted carrier may release its cargo entirely uncontrollably	[4,7,8,41]
Externally assisted delivery	Modification of a biological barrier by an external agent so that a co-administered drug can enter a compartment	The intervention acts on the tissue, not on the carrier	Counted as stimuli-responsive release, inflating the apparent clinical maturity of the field	[42]
Multi-stimuli responsiveness	Responsiveness to two or more cues, with no implication as to the logic relating them	Number of cues only	Used interchangeably with AND-gating (see Section 6.9 and Figure 7)	[25,26,34]
AND-gated release	Minimal release under either cue alone and substantial release only when both are present, all measured under identical conditions	Requires all four conditions and a pre-stated threshold for minimal activation	Asserted from dual-stimulus data alone, which cannot exclude OR, additive or cascade behavior	[25,26]

**Table 3:** Principal mathematical models used to describe drug release from nanoparticulate and hydrogel vehicles.

Model	Equation	Dominant Mechanism	Typical Application	Ref.
Zero-order	M_t_ = k_0_·t	Constant-rate release independent of drug remaining	Reservoir devices; surface-eroding matrices; and osmotic pumps	[27,28,44]
First-order	M_t_/M_∞_ = 1 − e^−k·t^	Release proportional to remaining drug	Dissolution-limited systems; liposomes in sink	[27,29]
Higuchi’s	M_t_/M_∞_ = k_H_·t^1/2^	Pure Fickian diffusion from a planar matrix	Matrix tablets; transdermal patches	[27,29]
Hixson–Crowell	M_0_^1/3^ − M_t_^1/3^ = k_s_·t	Surface area changes during dissolution	Erodible particulate systems	[27,29]
Korsmeyer–Peppas	M_t_/M_∞_ = k_KP_·t^n^	Geometry-dependent diffusion + relaxation; n discriminates Fickian (~0.43–0.50) vs. Case II (1.0)	Polymeric nanoparticles; swellable matrices; and nanogels	[27,29,47]
Peppas–Sahlin	M_t_/M_∞_ = k_1_·t^m^ + k_2_·t^2m^	Additive Fickian and relaxational contributions	Coated matrix tablets; multi-mechanism systems	[27,48]
Weibull	M_t_/M_∞_ = 1 − exp[−(t/τ)^β^]	Empirical/fractional-kinetic foundations	Heterogeneous systems with broad mechanism spectra	[27,28]
Super Case II	M_t_/M_∞_ = kKP·t^n^, *n* > 1 (slab)	Accelerating solvent front; relaxation plus stress-induced cracking or erosion	Highly swellable hydrophilic matrices; some nanogels	[27,43,44]

**Table 4:** Assumptions, validity domains and failure modes of the release models in Table 3.

Model	Key Assumptions	Valid Over	Fails When	Evidence Needed Beyond the Fit	Ref.
Zero-order	Constant active surface area; drug reservoir in excess; and sink maintained	Whole profile, while reservoir persists	The reservoir is exhausted, or surface area changes during release	Mass loss or geometric evidence that the releasing surface is constant	[27,44]
First-order	Rate proportional to remaining payload; no barrier other than dissolution	Whole profile	A diffusional or membrane barrier dominates, or a burst phase is present	Demonstration that dissolution, not diffusion, is rate-limiting	[27]
Higuchi’s model	Planar geometry; initial drug loading far above solubility; non-swelling, non-eroding matrix; constant diffusivity; and perfect sink	Approximately the first 60% of cumulative release	Applied to swelling, eroding, spherical or nanoscale systems, or fitted over the full curve	Gravimetric swelling and mass-loss data confirming that neither process contributes	[27,29,43]
Hixson–Crowell	Dissolution from particles of similar shape; surface area varies as the two-thirds power of mass	Whole profile for dissolving particulates	The particle does not dissolve but erodes or swells	Particle-size distribution over time	[27,29]
Korsmeyer–Peppas	Defined geometry; one-dimensional transport; constant boundary conditions; and the exponent is geometry-specific	Approximately the first 60–70% of cumulative release	Geometry is unstated; the full profile is fitted; and the released species is a particle rather than a molecule	Independent evidence of swelling or relaxation; identification of the released species (Section 5.2)	[27,29,43]
Peppas–Sahlin	Additive and separable Fickian and relaxational contributions	Initial-to-intermediate release	The two contributions are coupled rather than additive	Swelling kinetics measured independently of release	[27,48]
Weibull	Empirical distribution function; parameters have no assumed physical meaning	Whole profile	Interpreted mechanistically; the shape parameter is read as a transport regime	Treated as description only; mechanism must come from elsewhere	[27,28]
Modified first-order with burst term	A rapidly released surface-associated fraction superimposed on a slower first-order process	Whole profile for particulate release from nanocomposite matrices	The burst fraction is not separately characterized	Direct measurement of the escaping species, e.g., particle counting rather than total drug assay	[16,29]

**Table 5:** Comparative summary of the principal nanoparticulate platforms for controlled drug release.

Platform	Typical Size	Cargo Class	Loading Mechanism	Key Strengths	Key Limitations	Clinical Status	Typical Administration Route(s)
Liposome	50–200 nm	Hydrophilic + lipophilic	Aqueous core/bilayer partitioning	Approved since 1995; dual-cargo capacity	Drug leakage; storage stability	Multiple FDA/EMA approvals (Doxil^®^, Onivyde^®^, and AmBisome^®^) [7]	Intravenous
SLN/NLC	50–500 nm	Lipophilic (preferred)	Lipid matrix entrapment	GRAS lipids; scalable HPH; and ocular and dermal use	Limited loading for hydrophilic drugs	Cosmetic and topical products; clinical trials in oncology [57,58]	Topical, ocular, and oral
LNP (ionizable)	60–120 nm	Nucleic acids; small molecules	Electrostatic/hydrophobic	Industrial-scale mRNA and siRNA delivery	Cold-chain; immune reactivity at high dose	Onpattro^®^; Comirnaty^®^; and Spikevax^®^ [7,10,59]	Intravenous and intramuscular
Polymeric (PLGA)	100–300 nm	Small molecules; peptides; and proteins	Matrix dispersion	Tunable degradation; FDA/EMA-approved excipient	Acid-catalyzed bulk erosion; burst	Multiple long-acting injectables; >100 trials [45,46,60]	Subcutaneous, intramuscular, and intratumoral
Polymeric micelle	20–100 nm	Highly hydrophobic small molecules	Hydrophobic core sequestration	Solubilization; stimuli-responsive disassembly	CMC-related kinetic instability	Genexol-PM^®^ and Nanoxel^®^; NK105 in Phase III [61,62,63]	Intravenous
Dendrimer	1–15 nm	Small molecules; nucleic acids	Cavity encapsulation/surface conjugation	Monodisperse; precise stoichiometry	Cationic surface toxicity; cost	OP-101 (PAMAM-NAC), randomized phase 2a completed (NCT04458298); otherwise, preclinical [64,65]	Intravenous and intrathecal
Mesoporous silica	50–250 nm	Small molecules; biologics	Pore loading; surface ‘nanovalves’	High loading (>30%); gate-able release	Long-term in vivo silica clearance	Phase I (C-dot, FDA cleared as IND) [19,20]	Intravenous and intratumoral
Gold NP/nanorod	10–100 nm	Small molecules; oligonucleotides	Surface conjugation	Plasmonic NIR triggering; imaging	Non-biodegradable; long-term safety	Aurolase^®^ (Phase II; photothermal) [22]	Intratumoral and intravenous
Iron oxide/SPION	5–100 nm	Small molecules; biologics (in shell)	Polymer or lipid shell	MRI imaging; magnetic guidance; hyperthermia	Aggregation; macrophage uptake	Feraheme^®^ (anemia); NanoTherm^®^ (glioma; CE mark withdrawn) [23,66]	Intravenous, intratumoral
MOF	50–250 nm	Small molecules; biologics	Pore + coordination	Highest loading (up to 60% w/w); tunable	Stability in serum; metal toxicity concerns	Preclinical only [67,68,69]	Intravenous (preclinical)
Cell membrane coated	80–300 nm	Variable (core-dependent)	Core entrapment + surface targeting	Immune evasion; homotypic targeting	Membrane batch variability; regulatory novelty	Preclinical only [70,71,72]	Intravenous (preclinical)

**Table 6:** Comparative summary of the principal hydrogel platforms for sustained controlled release.

Platform	Polymer Examples	Crosslinking Mode	Key Strengths	Key Limitations	Representative Applications
Natural—polysaccharide	Alginate, chitosan, hyaluronic acid, and dextran	Ionic; pH; and physical entanglement	Biocompatibility; mucoadhesion; and ECM mimicking	Batch variability; mechanical weakness	Wound dressings; oral/intranasal delivery; and intra-articular HA [85,86,87]
Natural—protein	Collagen, gelatin (GelMA), fibrin, silk, and elastin	Photo-/thermal/enzymatic	Cell-instructive sequences; bioactivity	Immunogenicity risk; sterilization sensitivity	Tissue engineering; injectable depots [86,88]
Synthetic—covalent	PEG-diacrylate, PVA, and PHEMA	Covalent (photo, Michael, and click)	Reproducibility; tunable mechanics	Inert biology; possible reactive residues	Controlled-release implants; ophthalmic gels [12,86]
Thermo-responsive	PNIPAM, poloxamer 407, and ELP	LCST phase transition	In situ gelation at body temperature	Burst near LCST; PNIPAM clearance	Injectable depots; rectal/vaginal gels [12,89]
Supramolecular	Ad–CD; UPy–PEG; metal-coord.	Host–guest; H-bonding; and coordination	Shear-thinning; self-healing; and injectable	Lower mechanical strength	Intratumoral; intravitreal; and cardiac [11,89,90,91]
Polymer–nanoparticle (PNP)	HPMC-C12 + PEG-PLA NP	Hydrophobic NP–polymer	Months of release; broad cargo range	Carrier complexity; storage	Subcutaneous biologics; intravitreal [11,33]
Tough/DN/nanocomposite	DN PAMPS-PAAm; nanocomposite clay–PNIPAM	Combined sacrificial + permanent	Cartilage-like toughness; load bearing	Synthesis complexity	Cartilage repair; chronic wound dressings [86,92]
Microgel/nanogel	Cross-linked polysaccharides; PEG; and PNIPAM-co	Inverse emulsion; precipitation; and microfluidic	Stimuli-responsive; deformable; injectable	Polydispersity; scalability	Cancer chemo-immunotherapy; vaccines; and BBB delivery [30,93,94,95]
Granular/microgel-assembled	Jammed microgels (PEG, GelMA, and HA)	Inter-particle weak crosslinking	Interconnected porosity; cell infiltration	Mechanical fragility	Regenerative medicine; injectable scaffolds [11,17]

**Table 7:** Principal hybrid nanoparticle–hydrogel architectures and their characteristic release behavior.

Architecture	NP–Network Coupling	Release Mechanism	Mechanical Effect	Representative Applications	Released Species/Rate-Limiting Step
Free NP in bulk gel	Steric/weak adsorption	Drug diffusion + NP escape	Modest reinforcement	MSN-in-PEG and SPION-in-chitosan; antimicrobial wound dressings [12,16,18]	Dissolved drug and intact NP/mesh diffusion plus interfacial unbinding
Nanoparticle-crosslinked (PNP)	Multivalent dynamic (hydrophobic, host–guest, and boronate)	Erosion governed by crosslink unbinding	Shear thinning; self-healing	Intravitreal bimatoprost; subcutaneous biologic depots [11,33,98]	Dissolved drug and intact NP/crosslink unbinding and matrix erosion
Covalently tethered NP	Click/Michael/Schiff-base	NP retained; drug only released from NP	Strong; durable	Long-term implant coatings; theranostic constructs [11,15]	Dissolved drug only, NP retained/intra-particle release and linker cleavage
Microgel-in-gel/granular	Inter-particle weak crosslinking	Two-compartment; programmable	Porous; cell-infiltrable	Regenerative medicine; injectable scaffolds [11,17,30,95]	Dissolved drug and intact NP from two compartments/independent per compartment
Layered/stratified	Spatial gradient	Sequential exposure of layers	Engineered anisotropy	Microneedle patches; multi-layered depots [12,17]	Dissolved drug and matrix fragments/sequential layer erosion

**Table 8:** Stimuli-responsive chemistries: contrasts, kinetics and application contexts.

Stimulus	Responsive Moiety	Trigger Contrast (on/off)	Typical Response Time	Principal Applications
pH	Hydrazone, acetal, and ketal; charge-reversal amines	pH 7.4 → 5.0–6.5	Minutes (cleavage); seconds (charge)	Oncology; oral/intracellular [5,34,47,89,104]
Redox (GSH)	Disulfide, diselenide, and trisulfide	2 µM → 2–10 mM GSH	Seconds–minutes	Intracellular cancer therapy [105,106,107,108]
ROS	Thioketal, arylboronate, ferrocene, and selenium	~20 nM → 50–100 µM	Minutes	Tumor; inflammation [47,109,110,111]
Enzyme	MMP/HAase/cathepsin peptides	5–100× elevation	Minutes–hours	Tumor stroma; MI; and infection [5,47,112,113]
Hypoxia	Azobenzene, nitroaromatic, and 2-nitroimidazole	pO_2_ < 10 mmHg	Hours	Deep-tumor PDT–chemo combinations [47,114]
Temperature	PNIPAM, poloxamer 407, and ELP	<LCST → >LCST	Seconds	Injectable depots; magnetic/photothermal triggering [12,89,115]
Light	o-nitrobenzyl, azobenzene, and spiropyran; NIR-PT NPs; and UCNPs	Off → laser ON	Seconds	Skin, eye, and intra-operative; superficial tumors [22,116]
Magnetic	SPION + thermo-responsive shell	Field OFF → ON	Minutes	Deep-tumor; NanoTherm^®^ (CE marking discontinued; investigational) [23,66,117]
Ultrasound	Microbubbles; perfluorocarbon nanodroplets; and piezoelectric polymers	Acoustic OFF → ON	Seconds	BBB opening (barrier modulation); deep tissue [42,118]
Glucose	GOx; PBA-diol; and ConA-sugar	5 mM → 10–25 mM	Minutes	Diabetes; closed-loop insulin [21,31,32,119]
Multi-stimuli	Combined (e.g., pH + redox + light)	Two or more cues; the logic relating them (AND, OR, additive, sequential, and cascade) must be established separately (Section 6.9)	Variable	Precision oncology; reduced off-target [25,26,34]

**Table 9:** Stimulus-specific limitations: trigger variability, achievable activation thresholds and safety liabilities.

Stimulus	Trigger Variability in Patients	Clinically Achievable Activation	Principal Safety Liability	Ref.
pH	Tumor extracellular pH varies between and within lesions; overlaps with inflamed healthy tissue	Attainable; the endosomal–lysosomal gradient is reliable, while the extracellular one much less so	Premature activation in physiologically acidic compartments (stomach, inflamed joints, and ischemic tissue)	[5,34,47,104]
Redox (GSH)	Intracellular 1–10 mM; many published systems characterized at 50–100 mM	Attainable intracellularly; extracellular redox contrast is small	Depletion of physiological glutathione pools; off-target reduction in healthy cells	[105,106,107]
ROS	Lesion concentrations in the tens of micromolar against nanomolar baseline, but highly heterogeneous	Marginal for many linkers; frequently supplemented by exogenous ROS generation	Cleavage products (acetone and thiols from thioketals); iatrogenic oxidative stress where ROS is generated to drive release	[47,109,110,111]
Enzyme	5–100-fold overexpression, but activity depends on inhibitor balance, not expression alone	Attainable where the enzyme is extracellular and abundant	Cleavage by the same enzyme at physiological sites of expression	[5,47,112,113]
Hypoxia	Steep spatial gradients; the hypoxic fraction varies widely between tumors	Attainable only in the poorly perfused core, which is also the least accessible region	Reduction products of nitroaromatic and azo groups; overlap with physiologically hypoxic tissue	[47,114]
Temperature	Endogenous variation minimal; requires an applied source	Attainable with local hyperthermia at 40–43 °C	Thermal injury to adjacent tissue; unintended release during fever	[12,89,115]
Light	Operator controlled; off-state genuinely off	Penetration under 1 cm (visible) and 2–3 cm (near-infrared); restricted to skin, eye, oral cavity, intra-operative and superficial sites	Phototoxicity; thermal injury during photothermal conversion	[6,22,116]
Magnetic	Operator controlled	Deep-tissue access, but requires dedicated field-generating equipment	Thermal injury during hyperthermia; long-term fate of non-degradable iron oxide cores	[23,66,117]
Ultrasound	Operator controlled	Deep-tissue access with sub-millimeter focusing	Inertial cavitation and mechanical tissue damage; hemorrhage during barrier opening	[42,118]
Glucose	Physiological range is narrow (5 mM to 10–25 mM); the contrast is the smallest of any endogenous cue	Attainable but demanding, since a small input change must produce a large output change	Hypoglycemia from over-release; hydrogen peroxide generated by glucose oxidase; and concanavalin A immunogenicity	[21,31,32,119]
Inorganic carriers (all triggers)	Not applicable	Not applicable	Long-term persistence of non-degradable cores; metal-ion release from metal–organic frameworks and metal oxides	[19,20,66,69]
PEGylated carriers (all triggers)	Anti-PEG antibodies detectable in 50–90% of healthy adults	Not applicable	Accelerated blood clearance; hypersensitivity reactions	[123,124]

**Table 10:** Representative clinical-stage and approved nanoparticulate, hydrogel and hybrid formulations. Product type is reported separately from development stage, and the highest-level efficacy evidence is reported separately from current regulatory status, because these are not equivalent measures and the categories are not positions on a single continuum.

Therapeutic Area	Platform	Product Type	Drug/Cargo	Highest-Level Efficacy Evidence	Current Development or Regulatory Status	Ref.
Oncology	PEGylated liposome	Medicinal product	Doxorubicin (Doxil/Caelyx)	Randomized phase III	Approved (1995); multiple jurisdictions	[7]
Oncology	Liposome	Medicinal product	Irinotecan (Onivyde)	Randomized phase III (NAPOLI-1)	Approved; metastatic pancreatic adenocarcinoma	[7]
Oncology	Albumin-bound particle	Medicinal product	Paclitaxel (Abraxane)	Randomized phase III	Approved; breast, NSCLC, and pancreatic	[7]
Oncology	Polymeric micelle	Medicinal product	Paclitaxel (Genexol-PM; Nanoxel)	Randomized phase III (regional)	Approved in selected Asian jurisdictions	[61,62]
Oncology	Polymeric micelle	Medicinal product	Paclitaxel (NK105)	Phase III	Phase III	[61,62]
Oncology	Lipid nanoparticle	Medicinal product (individualized)	mRNA-4157/V940 plus pembrolizumab	Randomized phase 2b (KEYNOTE-942/mRNA-4157-P201): improved recurrence-free survival versus pembrolizumab alone	Phase 3, NCT05933577 (INTerpath-001), active, not recruiting; phase 3 also in NSCLC (NCT06077760)	[125,126]
Oncology	Iron oxide magnetic hyperthermia	Medical device	NanoTherm (no drug cargo)	Single-arm clinical series	CE-marked as a device 2010 for recurrent glioblastoma; sponsor insolvency 2022; CE marking discontinued; system currently investigational and not approved in any jurisdiction	[23,117,121]
Oncology	Gold nanoshell photothermal	Medical device/drug–device	Aurolase (no drug cargo)	Early-phase clinical	Phase II, prostate	[22]
Oncology	TLR3 agonist polyplex	Medicinal product	BO-112	Phase I with immune activation endpoint	Phase I/II combination trials	[127]
Endocrinology	Glucose-responsive microneedle patch	Preclinical responsive construct	Insulin	Preclinical (murine)	Preclinical	[21,31,32]
Endocrinology	Oral glucose-responsive nanoparticle	Preclinical responsive construct	Insulin	Preclinical (type 1 diabetes model)	Preclinical	[119]
Wound/regenerative	Multi-responsive nanocomposite hydrogel	Preclinical responsive construct	Antibiotic, dihydromyricetin, and photothermal	Preclinical (MRSA-infected diabetic wound)	Preclinical	[128]
Wound/regenerative	Sprayable exosome hydrogel	Preclinical responsive construct	MSC exosomes	Preclinical	Preclinical	[129]
Wound/regenerative	Catechol-quaternized chitosan/PEG hydrogel	Preclinical responsive construct	TGF-β3	Preclinical (diabetic model)	Preclinical	[130]
Wound/regenerative	Hyaluronic acid and silver dressings	Medical device	Antimicrobial	Clinical use	Marketed under the device pathway	[11,15]
Ocular	PNP injectable hydrogel	Preclinical responsive construct	Bimatoprost	Preclinical (rabbit); eight-week vitreal exposure, mild foreign-body response	Preclinical; no injectable hydrogel depot approved for ocular use	[33]
Ocular	NLC/SLN	Cosmeceutical and topical products	Topical antimicrobial, glaucoma agents	Varies by product	Marketed as cosmeceutical and topical formulations	[58]
CNS	Focused ultrasound plus microbubbles	Drug–device combination (barrier modulation)	Co-administered chemotherapy or antibody	Phase I/II feasibility	Phase I/II; opens the blood–brain barrier; does not itself constitute controlled cargo release (Section 2.1)	[42]
CNS/systemic inflammation	Hydroxyl-PAMAM dendrimer	Medicinal product	OP-101 (N-acetylcysteine conjugate)	Randomized phase 2a (PRANA, NCT04458298, n = 24) in severe COVID-19 hyperinflammation, with neuroinflammatory markers as secondary endpoints	Phase 2a completed; no phase III recorded for this indication	[131,132]
Intra-articular	Hyaluronic acid; PNP hydrogel	Medical device (HA); preclinical construct (PNP)	NSAIDs; biologics	Clinical (HA); preclinical (PNP)	HA marketed; PNP preclinical	[11,17]
Cardiovascular	MMP-responsive peptide–polymer micelle	Preclinical responsive construct	MMP inhibitor	Preclinical; 28-day retention in infarct	Preclinical	[113]
Cardiovascular	Monocyte-membrane-coated nanoparticle	Preclinical responsive construct	Verteporfin (YAP/TAZ inhibition)	Preclinical (murine atherosclerosis)	Preclinical	[133]
Cardiovascular	Hyaluronidase-responsive supramolecular assembly	Preclinical responsive construct	Aspirin and lovastatin; NIR-II photothermal	Preclinical; 53% reduction in plaque area in ApoE-deficient mice	Preclinical	[134]
Cardiovascular (atherosclerosis)	Protein-assembled EGCG/protamine nanoparticle, fucoidan-coated; protease- and H_2_O_2_-responsive	Preclinical responsive construct	Epigallocatechin gallate; nitric oxide from protamine	Preclinical (apolipoprotein-E-deficient mice); approximately 11.3-fold reduction in aortic lipid accumulation versus high-fat-diet control	Preclinical	[135]
Cardiovascular (restenosis)	ROS-responsive and ROS-scavenging cyclodextrin nanomedicine with collagen-IV-homing peptide	Preclinical responsive construct	Isosorbide dinitrate; ethyl caffeate	Preclinical (rat carotid balloon injury); largest reduction in intimal area and proliferation index at 14 days across six arms	Preclinical	[136]
Cardiovascular (aortic dissection)	Galectin-3-targeted, pH-, protease- and ROS-responsive pectin/protamine nanoparticle	Preclinical responsive construct	Resveratrol; nitric oxide from protamine	Preclinical (β-aminopropionitrile/angiotensin-II mice); dissection incidence from 87.5% to below 25%; four-week survival from 15% to 88%	Preclinical	[137]
Cardiovascular (abdominal aortic aneurysm)	ROS-responsive hyperbranched polylysine nanoparticle (phenylthio–sulfoxide switch)	Preclinical responsive construct	Selenomethionine	Preclinical (calcium chloride-induced aneurysm in mice); reduced aortic diameter, wall thickening and collagen deposition versus model and drug-free carrier	Preclinical	[138]
Cardiovascular (post-infarction)	ROS-responsive peptide–drug conjugate (boronate ester, 1,6-elimination)	Preclinical responsive construct	Isosorbide mononitrate	Preclinical (mouse left-anterior-descending ligation); improved ejection fraction and fractional shortening at 28 days	Preclinical	[139]
Cardiovascular (thrombosis)	MMP-2/9-responsive gelatin/fucoidan magnetic nanoprobe	Preclinical responsive construct	Urokinase; iron-oxide MRI contrast	Preclinical (rodent thrombus models); approximately 90% urokinase release within 2 h with MMP-9 in vitro	Preclinical; imaging function classified as diagnostic, not controlled release (Section 2.1)	[140]
Prophylactic vaccination (non-oncology)	Ionizable lipid nanoparticle	Medicinal product	mRNA (Comirnaty; Spikevax)	Randomized phase III	Approved; listed here because it demonstrates GMP nanomedicine manufacture at scale, not as an oncology product	[7,10,59]

**Table 11:** Registered clinical trials of nanoparticulate and hydrogel delivery systems discussed in this review. Registry entries verified against ClinicalTrials.gov, accessed on 2 August 2026.

Identifier	Intervention	Indication	Phase	Status at Access	Notes
NCT05933577 (INTerpath-001)	V940/intismeran autogene (mRNA-4157; lipid nanoparticle) plus pembrolizumab versus placebo plus pembrolizumab	Resected high-risk stage II–IV cutaneous melanoma	3	Active, not recruiting	Approximately 1089 participants; confirmatory trial for the phase 2b KEYNOTE-942/mRNA-4157-P201 efficacy signal [125,126]
NCT06077760 (INTerpath-002)	V940 (mRNA-4157) plus pembrolizumab	Resected stage II–IIIB non-small-cell lung cancer	3	Recruiting	Second phase 3 indication for the same individualized neoantigen platform
NCT04458298 (PRANA)	OP-101, hydroxyl-polyamidoamine dendrimer–N-acetylcysteine conjugate	Severe COVID-19 with hyperinflammation	2a	Completed	24 participants; single ascending intravenous dose; neuroinflammatory markers as secondary endpoints; results published in [131,132]

**Table 12:** Intellectual property landscape of the principal platform classes.

Platform Class	Representative Marketed Product(s)	Patent Position	Consequence for Translation	Product ref.
PEGylated liposome	Doxil/Caelyx (doxorubicin); Onivyde (irinotecan)	Foundational composition-of-matter claims expired; secondary claims on manufacturing and loading gradients	Generic and hybrid applications exist; approval now turns on demonstrating equivalence of a complex product, which returns to release test methodology (Section 2.3)	[7]
Albumin-bound particle	Abraxane (paclitaxel)	Core claims expired; process patents persist	Multiple follow-on products; the barrier is manufacturing reproducibility rather than freedom to operate	[7]
Polymeric micelle	Genexol-PM; Nanoxel (paclitaxel)	Regionally granted; block-copolymer composition claims	Approvals concentrated in the jurisdictions where the patents were prosecuted, which partly explains the regional pattern of authorization	[61,62]
Ionizable lipid nanoparticle	Onpattro; Comirnaty; and Spikevax	Dense and contested; ionizable lipid structures and microfluidic mixing processes heavily claimed	Freedom to operate, rather than formulation science, has been the limiting factor for several programs	[7,10,59]
PLGA long-acting depot	Multiple long-acting injectables	Core polymer claims long expired	Mature generic landscape; equivalence again depends on release test comparability	[45,46,60]
Iron oxide magnetic hyperthermia	NanoTherm (CE marking discontinued)	Device and method claims retained by the successor developer	Intellectual property survived the sponsor’s insolvency, but marketing authorization did not; a reminder that regulatory and commercial status are independent of patent status [121]	[23,117,121]
Stimuli-responsive hydrogel and hybrid constructs	None marketed	Predominantly platform and method-of-delivery claims rather than product claims	Consistent with preclinical status; commercial value currently resides in the delivery method rather than in a specific medicine	[11,15,17]

## Data Availability

No new data were created or analyzed in this study. Data sharing is not applicable to this article.
